# Systematic review of types of safety incidents and the processes and systems used for safety incident reporting in care homes

**DOI:** 10.1111/jan.16264

**Published:** 2024-06-19

**Authors:** Jason Scott, Kate Sykes, Justin Waring, Michele Spencer, Lesley Young‐Murphy, Celia Mason, Craig Newman, Katie Brittain, Pamela Dawson

**Affiliations:** ^1^ Northumbria University Newcastle upon Tyne UK; ^2^ Birmingham University Birmingham UK; ^3^ North Tyneside Community and Health Care Forum North Shields UK; ^4^ Newcastle University Newcastle upon Tyne UK; ^5^ Plymouth Marjon University Plymouth UK

**Keywords:** literature review, long‐term care, patient safety, quality of care

## Abstract

**Aims:**

To identify the safety incident reporting systems and processes used within care homes to capture staff reports of safety incidents, and the types and characteristics of safety incidents captured by safety incident reporting systems.

**Design:**

Systematic review following PRISMA reporting guidelines.

**Methods:**

Databases were searched January 2023 for studies published after year 2000, written in English, focus on care homes and incident reporting systems. Data were extracted using a bespoke data extraction tool, and quality was assessed. Data were analysed descriptively and using narrative synthesis, with types and characteristics of incidents analysed using the International Classification for Patient Safety.

**Data Sources:**

Databases were CINAHL, MEDLINE, PsycINFO, EMBASE, HMIC, ASSISA, Nursing and Allied Health Database, MedNar and OpenGrey.

**Results:**

We identified 8150 papers with 106 studies eligible for inclusion, all conducted in high‐income countries. Numerous incident reporting processes and systems were identified. Using modalities, typical incident reporting systems captured all types of incidents via electronic computerized reporting, with reports made by nursing staff and captured information about patient demographics, the incident and post‐incident actions, whilst some reporting systems included medication‐ and falls‐specific information. Reports were most often used to summarize data and identify trends. Incidents categories most often were *patient behaviour*, *clinical process/procedure*, *documentation*, *medication/intravenous fluids* and *falls*. Various contributing and mitigating factors and actions to reduce risk were identified. The most reported action to reduce risk was to improve safety culture. Individual outcomes were often reported, but *social/economic impact* of incidents and *organizational outcomes* were rarely reported.

**Conclusions:**

This review has demonstrated a complex picture of incident reporting in care homes with evidence limited to high‐income countries, highlighting a significant knowledge gap. The findings emphasize the central role of nursing staff in reporting safety incidents and the lack of standardized reporting systems and processes.

**Implications for the Profession and/or Patient Care:**

The findings from this study can inform the development or adaptation of safety incident reporting systems in care home settings, which is of relevance for nurses, care home managers, commissioners and regulators. This can help to improve patient care by identifying common safety issues across various types of care home and inform learning responses, which require further research.

**Impact:**

This study addresses a gap in the literature on the systems and processes used to report safety incidents in care homes across many countries, and provides a comprehensive overview of safety issues identified via incident reporting.

**Reporting Method:**

PRISMA.

**Patient or Public Contribution:**

A member of the research team is a patient and public representative, involved from study conception.

**What does this paper contribute to the wider global clinical community?**

- Provides the first global review of literature on safety incident reporting in care homes, providing recommendations for research including the need for research on safety incident reporting in low‐ and middle‐income countries.
- Identifies and reports on key safety issues in care homes that are currently identified via safety incident reporting, including patient behaviour, clinical processes and procedures, documentation, medication and intravenous fluids, and falls. The paper also identifies that social and economic outcomes are rarely captured in research which utilises safety incident reporting data.
- Highlights that nurses have a significant role to play in care home safety incident reporting, in keeping with their overall focus on safety work and safety leadership within care home settings.

## 1 INTRODUCTION

Incident reporting as a mechanism for learning from patient safety incidents is a well‐established part of healthcare delivery across a wide range of countries (Pham et al., 2010). There are numerous challenges to incident reporting, including debates around whether a deficit approach to safety promotes organizational learning (Waring et al., 2016), cultural factors that hinder the efficacy of incident reporting (Hamed & Konstantinidis, 2021), and whether safety incident reports alone are a sufficient way of identifying safety issues (Howell et al., 2017), instead of being recognized as a social process for learning (Macrae, 2016). Despite these challenges, the World Health Organization (WHO) states that an effective incident reporting system serves as a cornerstone of safe health practices and aids health care organizations in building a culture of safety (World Health Organization, 2005), defined as the shared values, beliefs and behavioural norms that contribute to the safety of patients (Feng et al., 2008). Safety incident reports can provide valuable information to support organizational learning though there is a need to ensure that there is a sense of ownership for the reporting system and that it needs to be embedded in other safety activities (Stavropoulou et al., 2015), again reflecting the importance of broader safety culture.

Safety incident reporting in care home settings, including residential and nursing homes (sometimes referred to as long‐term care facilities), is however in its relative infancy. Care homes themselves come in many forms depending on both the (inter)national context, with key differences between homes depending on their funding sources, regulatory systems and the type of care provided (Fischer et al., 2022). Differences between care homes exist both between and within different health and social care systems. There is an increasing amount of research examining incident reporting in care homes from various countries including the USA (Pierson et al., 2007; Wagner et al., 2008), Norway (Lafton & Fagerstrøm, 2011) and Australia (Tariq et al., 2012). Within care home settings, nurses play an important role in incident reporting and broader safety work (Johannessen et al., 2020; Prang & Jelsness‐Jorgensen, 2014), though a recent review of the literature which focused exclusively on incident reporting in care homes by nurses, did not attempt to classify the types of safety incidents that are identified in care homes nor did it examine the various types of incident reporting systems and processes used in care homes for capturing safety incident reports (Vaismoradi, Vizcaya‐Moreno, Jordan, Gåre Kymre, & Kangasniemi, 2020b). Furthermore, only five studies were included in the review, highlighting the fledgling nature of research on incident reporting in care homes.

This systematic review aimed to improve our understanding of safety incident reporting in care homes, with two specific objectives:

1. Identify the safety incident reporting systems and processes used within care homes to capture reports of safety incidents.
2. Identify the types and characteristics of safety incidents identified within care homes via safety incident reporting using the WHO International Classification for Patient Safety (Runciman et al., 2009; Sherman et al., 2009).

## 2 METHODS

The review was conducted in line with the PRISMA 2020 guidelines (Page et al., 2021). The review was not eligible for registration with PROSPERO, but the protocol was published elsewhere (Scott et al., 2022).

### 2.1 Database search

Seven electronic databases were searched in January 2023: CINAHL, MEDLINE, PsycINFO, EMBASE, HMIC, ASSISA and Nursing & Allied Health Database. In addition, grey literature was searched for using MedNar and OpenGrey. The first 100 hits from each of the grey literature databases were retrieved, which is deemed to be sufficient for grey literature searching (Godin et al., 2015; Haddaway et al., 2015). The Population, Interest, Context (PICo) framework was used to frame the search strategy, with Populations having no restrictions, Interest being incident reporting and Context being care homes. The full search strategy is provided in the Supplementary file S1.

### 2.2 Eligibility criteria

To be eligible for inclusion, studies had to be published in or after the year 2000, be original primary research written in the English language, focus on care homes (including residential and/or nursing homes), and incident reporting system(s), safety learning system(s), accident(s), and incident investigation system(s).

### 2.3 Study selection

Following the completion of the searches, all potentially eligible papers were imported into EndNote X9. One author (KS) screened all eligible titles and abstracts, with another author (JS) screening 20% of titles and abstracts. Discrepancies were resolved through discussion. There was an agreement rate of 96% (Cohen's Kappa = 0.66), equating to substantial agreement (McHugh, 2012). Following title and abstract screening, the full texts of studies were then double screened. One reviewer (KS) screened all full texts, with multiple reviewers (JS, CN, JW, PD, LYM and KS) independently screening a portion. Overall, there was an agreement rate of 77% (Cohen's Kappa = 0.53), equating to moderate agreement (McHugh, 2012). Disagreement at this stage was resolved through discussion with a third reviewer (JS). JS and KS had no disagreements at this stage, likely due to aligning their views during title and abstract screening.

### 2.4 Data extraction

A bespoke data extraction tool in Microsoft Excel was developed and independently piloted by two reviewers on six papers (three quantitative and three qualitative) (KS and JS). For all studies, the following data were extracted: author(s), year of publication, country of study, aim of the research, study design, methods and study setting, context of incident reporting, types of safety incident data reported, and systems/technology used to facilitate incident reporting. Quantitative study extraction also included study measures, type of analysis and results of descriptive and inferential statistics. Qualitative study extraction included data analysis, themes, quotes and a summary of findings. Mixed methods study extraction included a combination of all extracted properties. Full data extraction was conducted by one reviewer (KS), with one review (JS) reviewing six papers (two quantitative, two qualitative and two mixed methods papers). The data extracted between both reviewers were compared and discussed with discrepancies resolved.

### 2.5 Quality assessment

Each included study was assessed for quality using the McMaster critical appraisal tool (Law et al., 1998; Letts et al., 2007) or the mixed methods appraisal tool (MMAT) version 2018 (Hong et al., 2018). Two people (CM and CN) quality assessed all included papers. A third author (KS) checked a 10% sample of the included papers in each of the two categories (*n* = 17). Disagreements between ratings were agreed through discussion by reviewers.

### 2.6 Data synthesis

All studies included in this review focused on the systems and processes used for safety incident reporting, or characteristics of safety incidents captured via incident reports. For the systems and processes used for safety incident reporting, we generated a framework to focus on the methods used to report incidents, the process of reporting, who reports incidents, the types of data captured in incident reports, and the use of incident report data. Once coding was completed, we then narratively synthesized the data using the domains as the organizing principles. Narrative synthesis was used due to the heterogeneity of data (Popay et al., 2006). Characteristics of safety incidents were coded using a framework consisting of the WHO conceptual framework for the International Classification for Patient Safety (ICPS) (Runciman et al., 2009; Sherman et al., 2009) to identify the presence of data within several domains and sub‐domains within, including incident types, incident characteristics, detection of incidents, contributing factors, ameliorating actions, actions taken to reduce risk, mitigating factors and outcomes. One author (CM) coded the studies, with a second author (JS) checking a 10% sample of each of the two paper categories (*n* = 17). Studies could be coded several times within a specific domain and across domains.

## 3 RESULTS

**FIGURE 1:**
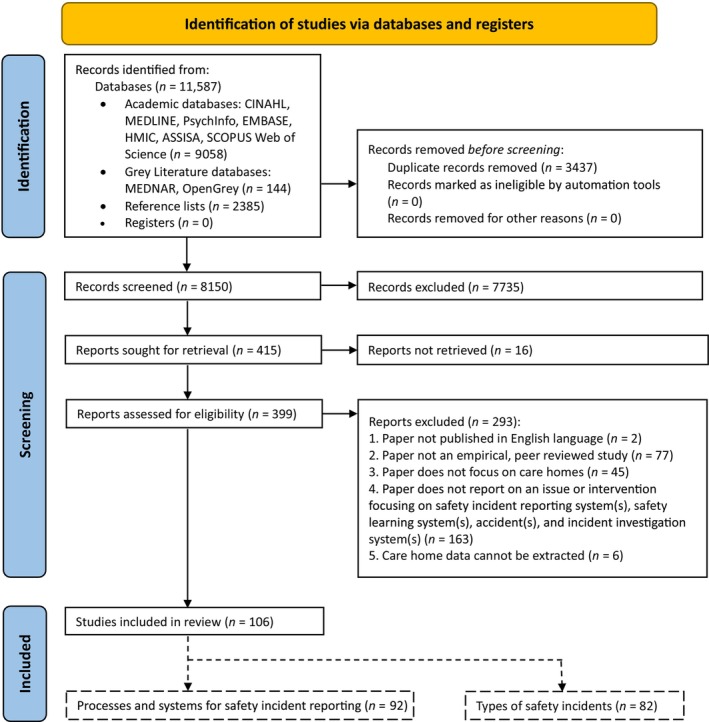
PRISMA flowchart.

A total of 11,587 articles were identified from the database search (Figure 1), of which 3437 were duplicates, leaving 8150 articles for screening. Following the review of title and abstract, 399 full‐text articles were retrieved for full‐text screening, with 106 meeting the inclusion criteria. Of these, 92 provided data on the processes and systems used for safety incident reporting in care home settings, and 82 provided data on the types of safety incidents in care home settings. All study references are included in relevant frequency tables in the corresponding sections. The results first present the characteristics of all 106 included studies followed by results related to safety incident reporting processes and systems, specifically the *process of reporting safety incidents*, *method(s) used to report safety incidents*, *people reporting safety incidents*, *how safety incident report data is used*, and *information captured by incident reporting systems*. Then, the *incident types* in care home settings are reported, including *categories of incident details*, *detection of incidents*, *mitigating factors*, *ameliorating actions*, *contributing factors* and *outcomes*. Throughout the sections, totals do not always equal the number of included studies as studies often reported on multiple processes, systems, incidents, factors and/or outcomes.

### 3.1 Study characteristics

**FIGURE 2:**
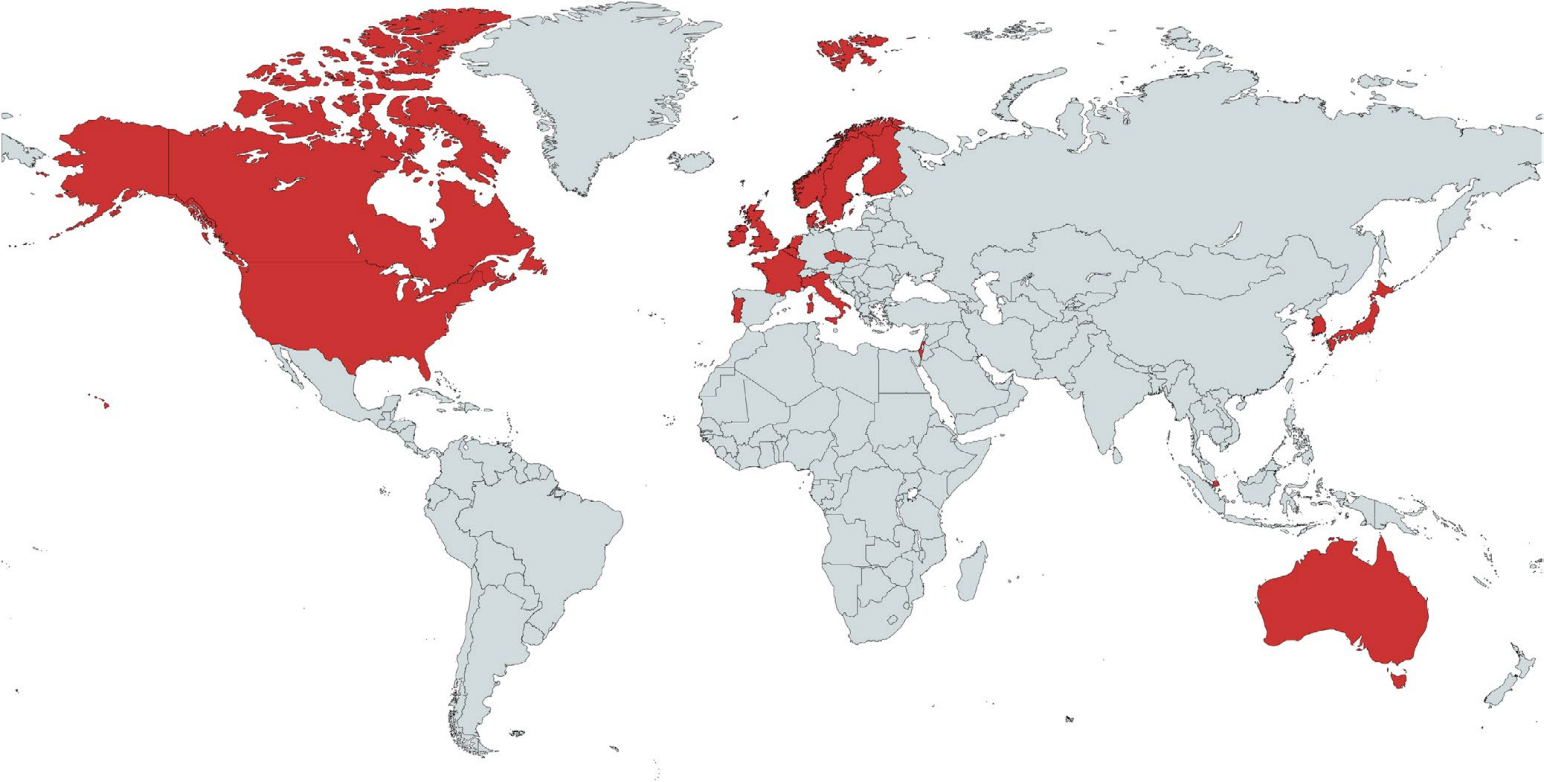
World map showing countries with studies included in the review.

From the 106 included papers, 81 (76.4%) were quantitative studies (Al‐Oraibi et al., 2012; Anderson et al., 2011; Andersson & Hjelm, 2017; Arain et al., 2016; Arfken et al., 2001; Baril et al., 2014; Barker et al., 2002, 2009; Brett et al., 2019; Capezuti et al., 2002, 2007; Chen et al., 2022; Crespin et al., 2010; Desai et al., 2011; Desai, Williams, Greene, Pierson, Caprio, & Hansen, 2013a, 2013b; Desai, Williams, Greene, Pierson, & Hansen, 2013c; DeSure et al., 2013; Dugré et al., 2021; Flynn et al., 2002; Francis‐Coad et al., 2018; Fuller et al., 2022; Gaskin et al., 2012; Gibson et al., 2008; Gil & Capelas, 2022; Gray‐Miceli et al., 2004, 2010; Greene et al., 2010; Gurwitz et al., 2005; Hansen et al., 2006, 2010; Hewitt et al., 2018; Hughes & Lapane, 2006; Jogerst et al., 2008; Joyce, 2020; Kapoor et al., 2019; Kepner et al., 2022; Kim et al., 2017; Kobayashi & Sugai, 2006; Kosse et al., 2015; Lachs et al., 2016; Lane, 2009; Lane et al., 2014; Lee & Cho, 2022; Liukka et al., 2021; Lord et al., 2003; Lundström et al., 2007; Mak et al., 2022; McCloskey et al., 2017; McDerby et al., 2019; Milligan, 2012; Milligan et al., 2011, 2014; Mirolsky‐Scala & Kraemer, 2009; Neyens et al., 2009; O'Regan et al., 2022; Pierson et al., 2007; Rahim‐Jamal et al., 2008; Rask et al., 2007; Ray et al., 2002; Robinovitch et al., 2013; Schuengel et al., 2020; Shmueli et al., 2014; Sjogren et al., 2015; Sluggett, Chen, et al., 2020; Sluggett, Hopkins, et al., 2020; Smith et al., 2022; Theodos, 2004; Tommasini et al., 2008; Toots et al., 2019; Verrue et al., 2011; Vlaeyen et al., 2021; Wabe, Seaman, et al., 2022; Wabe, Siette, et al., 2022; Wagner et al., 2005, 2008, 2012; Wagner, Castle, & Handler, 2013a; Whitney et al., 2017; Wilson et al., 2011; Yang et al., 2015), 15 (14.2%) were mixed methods studies (Blanchard et al., 2022; Carroll‐Solomon & Denny, 2005; Daly & Jogerst, 2005; Goh et al., 2021; Greene et al., 2005, 2011; Handler et al., 2004; Hrib et al., 2013; Johnson & Madan, 2017; Millet, 2013; St Clair et al., 2021; Vinther et al., 2017; Wagner, Castle, Reid, & Stone, 2013; Wagner, Damianakis, Pho, & Tourangeau, 2013; White, 2001) and 10 (9.4%) were qualitative studies (Ellis et al., 2012; McGrane et al., 2022; Myhre, Malmedal, Saga, Ostaszkiewicz, & Nakrem, 2020a; Myhre, Saga, Malmedal, Ostaszkiewicz, & Nakrem, 2020b; Picton et al., 2020; Prang & Jelsness‐Jorgensen, 2014; Serre et al., 2022; Storli et al., 2016; Tariq et al., 2012; Wagner et al., 2010). Nineteen countries (Figure 2) were represented, with over a third being based on research from the United States of America (*n* = 41, 38.7%) (Anderson et al., 2011; Arain et al., 2016; Arfken et al., 2001; Barker et al., 2002; Capezuti et al., 2002, 2007; Carroll‐Solomon & Denny, 2005; Chen et al., 2022; Crespin et al., 2010; Daly & Jogerst, 2005; Desai et al., 2011; Desai, Williams, Greene, Pierson, Caprio, & Hansen, 2013a, 2013b; Desai, Williams, Greene, Pierson, & Hansen, 2013; DeSure et al., 2013; Flynn et al., 2002; Gray‐Miceli et al., 2004, 2010; Greene et al., 2005, 2010, 2011; Gurwitz et al., 2005; Handler et al., 2004; Hansen et al., 2006, 2010; Hughes & Lapane, 2006; Jogerst et al., 2008; Kapoor et al., 2019; Kepner et al., 2022; Lachs et al., 2016; Lane, 2009; Lane et al., 2014; Mak et al., 2022; Millet, 2013; Mirolsky‐Scala & Kraemer, 2009; Pierson et al., 2007; Rask et al., 2007; Ray et al., 2002; Theodos, 2004; Wagner, Castle, & Handler, 2013; Wagner, Castle, Reid, & Stone, 2013), followed by Australia (*n* = 20, 18.9%) (Barker et al., 2009; Brett et al., 2019; Dugré et al., 2021; Francis‐Coad et al., 2018; Gaskin et al., 2012; Gibson et al., 2008; Hewitt et al., 2018; Joyce, 2020; Lord et al., 2003; McDerby et al., 2019; Picton et al., 2020; Sluggett, Chen, et al., 2020; Sluggett, Hopkins, et al., 2020; Smith et al., 2022; St Clair et al., 2021; Tariq et al., 2012; Wabe, Seaman, et al., 2022; Wabe, Siette, et al., 2022; White, 2001; Wilson et al., 2011), Canada (*n* = 15, 14.2%) (Baril et al., 2014; Ellis et al., 2012; Fuller et al., 2022; Johnson & Madan, 2017; Kim et al., 2017; McCloskey et al., 2017; Rahim‐Jamal et al., 2008; Robinovitch et al., 2013; Serre et al., 2022; Wagner et al., 2005, 2008, 2010, 2012; Wagner, Damianakis, Pho, & Tourangeau, 2013; Yang et al., 2015), and United Kingdom (*n* = 5, 4.7%) (Al‐Oraibi et al., 2012; Milligan, 2012; Milligan et al., 2011, 2014; Whitney et al., 2017). The quality of studies ranged from 4 (poor) to 16 (excellent), with a mean score of 10 (fair). Sixty‐eight (64.2%) studies reported the number of incidents analysed. Study characteristics are reported in full in Table 1.

**TABLE 1:** Characteristics of included studies.

Authors (publication year)	Country	Study design and methods	Participants/description of data sources	Number of incidents/reports related to care homes	Number and description of care homes included	Quality score
Al‐Oraibi et al. (2012)	UK	Quantitative	Incidence of falls, identified through a review of incident reports before and after assistive technology system installed.	Care home 1: 150 falls before intervention, zero falls reported 6 months after Care Home 2: 52 falls before intervention, 72 falls reported 6 months after	Two homes. One was a residential and rehabilitation‐focused home, accommodating up to 38 older people, and the second accommodated 32 older people with advanced dementia	8; Poor
Anderson et al. (2011)	USA	Quantitative	1286 participants (1286 nurses).	Not reported	109 licensed nursing homes	8; Poor
Andersson and Hjelm (2017)	Sweden	Quantitative	Reports from nursing homes submitted to Health and Social Care Inspectorate. The sample consists provider's written description of the serious adverse events and what they thought were the contributing factors. The reports were typed and written in free text and included the classification of the serious adverse events.	173 reports	Not reported.	9; Fair
Arain et al. (2016)	USA	Quantitative	Error reports submitted by staff in long‐term care facilities.	218 reports	Four long‐term care facilities; two control homes	8; Poor
Arfken et al. (2001)	USA	Quantitative	Pharmacy records, medical records, fall logs and incidence reports for one nursing home were obtained.	447 falls by 221 different residents	One nursing home with 180 beds	8; Poor
Baril et al. (2014)	Canada	Quantitative	Error reports submitted by staff to a voluntary error reporting system.	798 error reports before intervention, 2289 error reports after intervention	Six nursing homes covering 800 patients	12; Good
Barker et al. (2002)	USA	Quantitative	Observations of staff preparing and administering 50 doses by nurses. The observer wrote down what happened, information about the resident and medication.	Not reported	Six skilled‐nursing facilities	9; Fair
Barker et al. (2009)	Australia	Quantitative	87 participants (87 nursing home residents) Falls were determined by researchers reviewing medical notes and incident reports for falls within the 6‐month period following completion of the baseline assessment	Not reported	Six residential aged‐care facilities	10; Fair
Blanchard et al. (2022)	France	Mixed methods	20 participants (6 registered nurses, 14 nursing assistants)	76 incidents reports	2 long‐term care facilities (nursing home contains three conventional units for 94 residents [respectively, 31, 31 and 32 residents per unit] and a special care unit for people with dementia for 26 residents with challenging behaviours)	10; Fair
Brett et al. (2019)	Australia	Quantitative	55 participants (55 nursing home residents) Falls incidents were identified from nursing home incident reports	Before intervention; Intervention 1: median = 0; Intervention 2: median = 0; control: median = 0 After intervention; Intervention 1: median = 0; Intervention 2: median = 0; control: median = 1	Two nursing homes operated by the same company. One had 98 residents, the second had 101. 88% of residents in the first home were living with dementia, compared with 72% in the second	15; Excellent
Capezuti et al. (2002)	USA	Quantitative	463 participants (463 residents)	Not reported	Three not‐for‐profit nursing homes	14; Very good
Capezuti et al. (2007)	USA	Quantitative	251 participants (251 residents)	Restrictive side rail use Baseline (*n* = 710) Immediately post‐intervention (*n* = 719) One year post‐intervention (*n* = 707)	Four medium‐sized urban nursing homes. Mean bed size is 183. Three not‐for‐profit nursing homes and one for‐profit	13; Very good
Carroll‐Solomon and Denny (2005)	USA	Mixed methods	Implementation of a Quality Improvement System. Reports submitted using system were reviewed.	3500 events and near misses reported	18 long‐term care facilities that are part of a Catholic health system	5; Poor
Chen et al. (2022)	USA	Quantitative	Hospital admissions, skilled nursing facility claims, and facility information were extracted from the Certification and Survey Provider Enhanced Reporting (CASPER).	60,203 skilled‐nursing facility claims with a primary diagnosis of pressure ulcer	Not reported	10; Fair
Crespin et al. (2010)	USA	Quantitative	Self‐report data based on the Medication Error Quality Initiative–Individual Error (MEQI‐IE) reporting system during 2007 and 2008. MEQI‐IE is a web‐based reporting system that guides users through a step‐by‐step process to document each error incident and prompts users with specific questions depending on the type of error.	15,037 incidents reports	294 nursing homes	8; Poor
Daly and Jogerst (2005)	USA	Mixed methods	594 participants (289 administrators, 305 directors of nursing)	385 incidents	409 Medicare‐certified independent nursing homes	8; Poor
Desai et al. (2011)	USA	Quantitative	Self‐report data based on the MEQI‐IE reporting system fiscal years 2007 to 2009.	27,759 incidents reports	394 nursing homes. 73% had chain ownership and 77% had for‐profit operations.	8; Poor
Desai, Williams, Greene, Pierson, Caprio, and Hansen (2013a)	USA	Quantitative	Self‐report data based on the MEQI‐IE reporting system during fiscal years 2010 to 2011 and analysis of National Nursing Home Survey data.	32,176 incidents reports	396 nursing homes. Mean number of beds per nursing home was 120. 73% had chain ownership, and 77% were for profit.	8; Poor
Desai, Williams, Greene, Pierson, Caprio, and Hansen (2013b)	USA	Quantitative	Self‐report data based on the MEQI‐IE reporting system during fiscal years 2010 to 2011.	32,176 incidents reports	396 nursing homes. Mean number of beds per nursing home was 120. 73% had chain ownership, and 77% were for profit.	7; Poor
Desai, Williams, Greene, Pierson, and Hansen (2013)	USA	Quantitative	Self‐report data based on the MEQI‐IE reporting system during fiscal years 2010 to 2011.	32,176 incidents reports	396 nursing homes. The mean number of beds was 120. 73% had chain ownership, and 77% were for profit.	8; Poor
DeSure et al. (2013)	USA	Quantitative	27 participants (27 residents) Falls were determined by records routinely kept by the facility	Intervention: 7 falls Control: 10 falls	1 98‐unit assisted living community	14; Very good
Dugré et al. (2021)	Australia	Quantitative	242 participants (242 residents)	148 medication incident reports	8 residential aged‐care facilities	16; Excellent
Ellis et al. (2012)	Canada	Qualitative	22 participants (22 nurses)	Not reported	Two long‐term care facilities. Both were not‐for‐profit and together housed over 600 residents.	15; Excellent
Flynn et al. (2002)	USA	Quantitative	Comparison of incident reports, chart review and direct observation for reporting errors.	457 errors	36 hospitals and skilled‐nursing facilities (the breakdown of how many care homes within the total sample not reported).	10; Fair
Fuller et al. (2022)	Canada	Quantitative	Paper‐based reports of medication incidents that were voluntarily submitted.	270 medication incidents reports	1 long‐term care facility (239‐bed)	9; Fair
Francis‐Coad et al. (2018)	Australia	Quantitative	3015 participants (3015 residents)	3819 admissions	13 residential aged‐care sites. The mean number of beds was 60	11; Good
Gaskin et al. (2012)	Australia	Quantitative	119 participants (9 managers/health informatics officers, 23 registered nurses, 21 enrolled nurses/endorsed enrolled nurses, 66 assistants in nursing)	Not reported	Four residential aged‐care facilities. The mean number of residents was 142	10; Fair
Gibson et al. (2008)	Australia	Quantitative	Audit of residents' charts to identify ‘incident records’, which report details of fall incidents.	4870 falls	98 aged‐care facilities. 42 were classed as low care, 24 high care and 22 mixed. The highest number of beds in a facility was 136	7; Poor
Gil and Capelas (2022)	Portugal	Quantitative	150 participants (150 care workers)	Not reported	16 care homes (12 not‐for‐profit and 4 for profit nursing homes). On average, there were 36 residents in the nursing homes surveyed, varying between 9 and 84 residents, providing care to physically and mentally dependent people	10; Fair
Goh et al. (2021)	Singapore	Mixed methods	Cycle 1–42 participants (42 nurses) Cycle 2–21 participants (21 nurses).	Not reported	One nursing home with 624 beds	4; Poor
Gray‐Miceli et al. (2004)	USA	Quantitative	Director of long‐term care facilities provided a copy of any post‐fall evaluation/assessment tools used.	Not reported	149 long‐term care facilities	6; Poor
Gray‐Miceli et al. (2010)	USA	Quantitative	Facility‐level fall data from incident reports for the pre‐ and post‐intervention and all fall incident reports from monthly fall summary sheet.	Falls: Pre‐intervention (*n* = 286) Intervention (*n* = 207) Post‐intervention (*n* = 307)	One retirement community providing 110 beds of assisted living and skilled nursing in four units	13; Very good
Greene et al. (2005)	USA	Mixed methods	Pilot: submit 6 months' worth of medication error data. Follow‐up survey: Data entry of medication errors onto web‐based annual report form; Follow‐up survey on impact of report.	10,920 errors reported	Pilot: 10 nursing homes Main data entry for year: 385 nursing homes Follow‐up survey: 90 nursing homes	7; Poor
Greene et al. (2010)	USA	Quantitative	Nursing homes submit medication error reports as they occurred during the year using an optional web‐based reporting tool from 1 October 2006 to 30 September 2007.	5823 incident reports	203 nursing homes	8; Poor
Greene et al. (2011)	USA	Mixed methods	Pilot: 6‐month pilot to test the graphic reports Interviews: Two participants (one from each site).	Not reported	15 nursing homes	8; Poor
Gurwitz et al. (2005)	USA	Quantitative	1247 participants (1247 residents)	815 errors reported	Two large academic long‐term care facilities. The two facilities have a total of 1229 beds.	7; Poor
Handler et al. (2004)	USA	Mixed methods	Interviews: One participant (one director of nursing) Survey: 20 participants (6 administrative nursing staff, 14 clinical nursing staff) Review of medication error reports.	88 medication error reports	One long‐term care facility that was non‐profit with 126 beds	9; Fair
Hansen et al. (2006)	USA	Quantitative	Summary reports of medication errors submitted by nursing homes from 1 January 2004 to 30 September 2004 using a Web‐based reporting system.	9272 medication errors	384 nursing homes	8; Poor
Hansen et al. (2010)	USA	Quantitative	Individual reports of incidents, near misses and circumstances of unsafe conditions submitted to MEQI.	6459 reports	229 homes. Average size is 119 beds and the majority of homes are located in metropolitan areas, operated for profit and part of a chain	7; Poor
Hewitt et al. (2018)	Australia	Quantitative	221 participants (221 residents) Falls were measured by auditing incident records kept as standard practice in all facilities	Intervention: 142 falls Control: 277 falls	16 residential aged‐care facilities	15; Excellent
Hrib et al. (2013)	Czech Republic	Mixed methods	Survey: 128 participants (128 facility directors) Interviews: 63 facilities (description of participants not provided, covered nursing homes, mental hospitals, hospice, rehabilitation hospitals and other services).	128 incidents reported	32 nursing homes (in addition to the nursing homes, 11 mental hospitals, seven hospices, six rehabilitation hospitals and 11 other facilities were involved in the study)	7; Poor
Hughes and Lapane (2006)	USA	Quantitative	1003 participants (367 nurses and 636 nursing assistants)	Not reported	26 nursing homes	7; Poor
Jogerst et al. (2008)	USA	Quantitative	Incident reports filed by nursing home staff and complaints filed by persons other than service providers obtained from the federal complaints/incidents tracking system.	8308 incidents reports	Not specifically reported, but across 43 states	7; Poor
Johnson and Madan (2017)	Canada	Mixed methods	52 participants (18 nurses, 14 directors of care, 12 directors of nursing, 8 directors of resident care and 5 registered nurse supervisors)	Not reported	52 long‐term care facilities. 28 were in rural settings, 24 in urban. 36 were municipally owned and not‐for‐profit, while 16 were privately run and for‐profit. The mean number of beds was 85	7; Poor
Joyce (2020)	Australia	Quantitative	Incidents were extracted from the organizational incident reporting system.	313 incident reports assessed, of which 169 were analysed	13 aged‐care facilities. All operated by a single, not‐for‐profit provider; 9 metropolitan (*n* = 9) and 4 regional. Bed sizes ranged from 20 to 150 beds (median 54; mean 69.5), with a total bed capacity of 903	10; Fair
Kapoor et al. (2019)	USA	Quantitative	555 participants (555 residents). Adverse event within the 45‐day period after transition from hospital back to nursing home	716 adverse events	32 nursing homes. 24 homes were for profit, with 7 being not‐for‐profit. The mean number of beds was 130	8; Poor
Kepner et al. (2022)	USA	Quantitative	Reports submitted to the Pennsylvania Patient Safety Reporting System (PA‐PSRS) collecting healthcare‐associated infection reports from 375,000 long‐term care facilities.	17,971 infection reports	Not reported	8; Poor
Kim et al. (2017)	Canada	Quantitative	724 participants (724 residents). Incident reports of aggressive events occurring within 30 days of admission were collected.	53 incidents of one or more aggressive behaviours	25 long‐term care facilities	8; Poor
Kobayashi and Sugai (2006)	Japan	Quantitative	Review of incident reports collected from November 2002 to January 2003.	247 incident reports	Six nursing homes. Two nursing homes were small (30–40 residents), one nursing home was medium‐sized (90–150 residents), and three homes were large (200–400 residents)	7; Poor
Kosse et al. (2015)	Denmark	Quantitative	20 participants (20 long‐term care residents with dementia) Falls were documented on structured fall incident forms	115 falls	One long‐term residential care facility, 20 beds	10; Fair
Lachs et al. (2016)	USA	Quantitative	407 cases of resident‐to‐resident aggression	Zero incident reports indicated resident‐to‐resident aggression	Ten homes (five urban, five suburban)	6; Poor
Lane (2009)	USA	Quantitative	Self‐report data based on the MEQI‐IE reporting system during fiscal year 2007	13,551 errors reported	423 skilled‐nursing facilities. 43 were not‐for‐profit, 246 were for profit and 4 were Government owned, 293 were chain affiliated.	7; Poor
Lane et al. (2014)	USA	Quantitative	Self‐report data based on the MEQI‐IE reporting system from 1 October 2006 to 30 September 2007	581 errors reported	137 skilled‐nursing facilities. The mean number of beds was 120. 39 were not‐for‐profit, 98 were for profit. 93 were chain affiliated. 85 of the 137 were in an urban location.	8; Poor
Lee and Cho (2022)	South Korea	Quantitative	159 participants (88 registered nurses, 71 nurse aids)	Not reported	33 long‐term care facilities	10; Fair
Lord et al. (2003)	Australia	Quantitative	All residents were followed up until death or for a period of at least 6 months. Falls were ascertained from incident reports and medical records (examined every 6–12 weeks).	2554 falls	26 nursing homes and 17 intermediate‐care residences	10; Fair
Liukka et al. (2021)	Finland	Quantitative	196 participants (7 manager. 37 registered nurses, 152 practice nurses and 3 others. Note: double counting may be present as there were 196 participants who documented their gender) Data from the organizations' incident reporting system to determine the number of reported patient safety incidents reported.	Not reported	Nursing homes and long‐term wards with 888 beds	9; Fair
Lundström et al. (2007)	Sweden	Quantitative	149 participants (6 registered nurses, 13 assistant nurses, 101 nurse aides)	Not reported	10 homes for people with learning disabilities. 65 residents across the homes	9; Fair
Mak et al. (2022)	USA	Quantitative	148 participants (148 residents living with dementia). Falls incidents were collected from the incident management system.	Not reported	16 long‐term care residences	13; Very good
McCloskey et al. (2017)	Canada	Quantitative	Retrospective data from reported staff incidents and prospective data from 360 h of staff observations.	898 incidents reported	Five long‐term care facilities. All facilities were Government regulated, publicly funded and not‐for‐profit	7; Poor
McDerby et al. (2019)	Australia	Quantitative	Residential care pharmacist position implemented at study site for 6 months to perform medication reviews and quality improvement activities. Observational audits of medication rounds were performed, and documentation relating to allergies, adverse drug reactions and medication incidents was obtained from both sites before and after the pharmacist trial period. Study site: 74 participants (74 residents) Control site: 43 participants (43 residents).	Study site: pre‐intervention‐80 incidents, post‐intervention‐154 incidents Control site: pre‐intervention‐31 incidents, post‐intervention‐52 incidents	Two residential aged‐care homes; both homes belonged to the same organization. Study site had 104 beds, and the control site had 100 beds	10; Fair
McGrane et al. (2022)	Ireland	Qualitative	Safety incidents notification from residential care facilities across Ireland, submitted to the Database of Statutory notification from Social Care in Ireland. Notifications of unexpected death, infectious disease outbreak, serious injury to residents, unexplained absence of residents, allegations of abuse, staff misconduct, professional review of members of staff and, fire, loss of service or unplanned evacuation were documented.	14,611 notifications	1764 residential care facilities	10; Fair
Millet (2013)	USA	Mixed methods	Freedom of Information Act request of medication incidents in care home setting through analysis of voluntary incident reports submitted by NHS staff to the National Reporting and Learning Service from 1 January 2005 to 31 December 2009.	768 incident reports	Not reported	7; Poor
Milligan (2012)	UK	Quantitative	A Freedom of Information request was made to the NRLS, between 1 January 2005 and 31 December 2009.	768 incidents	Not reported	6; Poor
Milligan et al. (2011)	UK	Quantitative	Freedom of Information request made of medication incidents in the care home setting through analysis of voluntary incident reports submitted by NHS staff to the National Reporting and Learning Service (NRLS) from 1 January 2005 to 31 December 2009.	768 incidents	Not reported	6; Poor
Milligan et al. (2014)	UK	Quantitative	Three case studies of the types of incidents reported from incidents reported by hospital staff who became aware of medication errors that had occurred to patients discharged to the nursing home setting.	3 incident reports	76 care homes (homes provided nursing care, with a portion having both residential and nursing care)	7; Poor
Mirolsky‐Scala and Kraemer (2009)	USA	Quantitative	One 85‐year‐old female with Alzheimer's disease, completed a 4‐week physical therapy fall management programme consisting of 12 × 30‐min sessions.	Pre‐intervention: two incidents Post‐intervention: zero incidents	1 long‐term care facility	8; Poor
Myhre et al. (2020a)	Norway	Qualitative	43 participants (15 nursing home directors, 28 ward leaders)	Not reported	21 nursing homes covering six municipalities	10; Fair
Myhre et al. (2020b)	Norway	Qualitative	43 participants (15 nursing home directors, 28 ward leaders)	Not reported	21 nursing homes covering six municipalities	11; Good
Neyens et al. (2009)	Netherlands	Quantitative	518 participants (518 residents) Data on falls were collected prospectively by asking all participating wards to keep records of any fall incident on a structured report form	Intervention: 355 falls Control: 422 falls	12 nursing homes	15; Excellent
O'Regan et al. (2022)	Ireland	Quantitative	11 participants (5 residents from long‐term care facilities for older people; 6 residents from long‐term care facilities for people with disability) Notification from residential care facilities across Ireland, submitted to the Database of Statutory notification from Social Care in Ireland.	95,231 notifications	Long‐term care facilities (number not reported)	10; Fair
Picton et al. (2020)	Australia	Qualitative	Focus group: 32 participants (roles not reported) Interview: 14 participants (1 health service executive, 1 General practitioner on Medication advisory committee [MEC], 2 General practitioner not on MEC, 1 Geriatrician, 2 Community Pharmacists supplying Residential aged‐care services, 2 Hospital pharmacists, 1 National Prescribing Service pharmacist, 2 Consultant pharmacist on MEC, 1 RN not on MEC, 1 EN not on MEC)	Not reported	Four regional and rural health services that operated 27 public‐sector Residential aged‐care services	11; Good
Pierson et al. (2007)	USA	Quantitative	Errors occurred during a one year period was entered into a web‐based reporting system, and then completed an evaluation survey was conducted to assess usability and the potential for the system to prevent errors.	631 error reports	23 nursing homes	7; Poor
Prang and Jelsness‐Jorgensen (2014)	Norway	Qualitative	13 participants (13 nurses)	Not reported	17 nursing homes covering three municipalities	11; Good
Rahim‐Jamal et al. (2008)	Canada	Quantitative	Phase 1—Retrospective chart review where a physician‐pharmacist team independently reviewed the charts of 40 residents for a 12‐month period to understand under reporting. Phase 2—Four physicians and a nurse practitioner followed up 160 patients over 5 months, and systematically reported adverse drug reactions.	Phase 1–16 incidents reported Phase 2–33 incidents reported	Phase 1—Not reported. Phase 2—Five long‐term care facilities	7; Poor
Rask et al. (2007)	USA	Quantitative	Residents at nursing homes completed a fall management programme, between September 2004 and September 2005.	Not reported	Intervention: 19 nursing homes Non‐intervention: 23 nursing homes	10; Fair
Ray et al. (2002)	USA	Quantitative	2510 residents identified as using antidepressants and having falls.	Not reported	53 nursing homes	8; Poor
Robinovitch et al. (2013)	Canada	Quantitative	130 participants (130 residents).	227 incidents of falls	Two long‐term care facilities. One home has a 312‐bed, for‐profit facility; the second has 236‐bed and is a not‐for‐profit	7; Poor
Schuengel et al. (2020)	Netherlands	Quantitative	Incident reports collated from 5 September 2016 to 25 June 2020	Not reported	One large long‐term care organization for people with intellectual disabilities that operates about 1000 locations divided across 25 regions	8; Poor
Serre et al. (2022)	Canada	Qualitative	9 participants (9 nurses)	Not reported	3 long‐term care homes. All not‐for‐profit, between 120 and 160 beds across 4 to 5 units	9; Fair
Shmueli et al. (2014)	Israel	Quantitative	Adverse event reports collected from the various departments of the geriatric centre from 2008 to 2010.	1364 reports of adverse events	One large geriatric centre	8; Poor
Sjogren et al. (2015)	Sweden	Quantitative	39,111 participants (39,111 residents) Events that are reported at a provider of domiciliary dental care from 1 May 2012 to 30 June 2014.	724 reported events	Not reported	7; Poor
Sluggett, Hopkins, et al. (2020)	Australia	Quantitative	242 participants (242 residents) Medication administration times were ascertained from data extracted from resident medication administration charts. Falls were extracted from the provider's risk management and reporting software. Falls were reported prospectively by staff according to the organization's Client Incident Reporting Policy	Intervention: Baseline = 300 falls; Follow‐up = 410 falls. Comparison: Baseline = 421 falls; Follow‐up = 258 falls.	Eight residential aged‐care facilities	14; Very good
Sluggett, Chen, et al. (2020)	Australia	Quantitative	242 participants (242 residents) Details of all fall and medication incidents within the residential aged‐care facilities before and after study entry were extracted from the risk management system maintained by the aged‐care organization.	Not reported	Eight residential aged‐care facilities	14; Very good
Smith et al. (2022)	Australia	Quantitative	53 participants (53 residential aged care nurses)	6 reports	Residential age care services (number not reported)	8; Poor
St Clair et al. (2021, 2022)	Australia	Mixed methods	Deidentified data was extracted from an electronic management system. Information on adverse incidents was sourced from Cintellate which is an environment, health and safety software package.	60,268 reports	72 residential aged‐care facilities	10; Fair
Storli et al. (2016)	Norway	Qualitative	29 participants (16 nurses and 13 student nurses), and 69 learning logs	18 out of 69 learning logs included the handling of medication	Two nursing homes: one large urban nursing home with over 100 places distributed amongst three large wards, and one rural nursing home with approximately 60 places distributed amongst five small wards. A third nursing home's learning logs were included. No further information provided	12; Good
Tariq et al. (2012)	Australia	Qualitative	23 participants (18 staff from residential care facilities, 5 pharmacy staff), and 62 h of observations (50 h from residential care facilities and 12 from pharmacy)	Not reported	Three residential aged‐care facilities, non‐profit organization	14; Very good
Theodos (2004)	USA	Quantitative	Post‐fall intervention programme to reduce falls, which were then followed up with post‐fall assessment and incident reporting.	173 incidents of falls	Not reported	6; Poor
Tommasini et al. (2008)	Italy	Quantitative	112 participants (71 patients in hospital and 41 residents).	135 incident reports	Three nursing homes (as well as one hospital)	8; Poor
Toots et al. (2019)	Sweden	Quantitative	186 participants (186 residents) Data on falls during the intervention and follow‐up period were collected by review of fall incident reports in electronic medical records at nursing homes	Intervention: 6 month follow‐up = 111 falls; 12 month follow‐up = 232 falls Control: 6 month follow‐up = 113 falls; 12 month follow‐up = 241 falls	16 nursing homes	15; Excellent
Verrue et al. (2011)	Belgium	Quantitative	188 participants (76 facility directors, 112 nurses)	Not reported	76 nursing homes. The mean number of beds was 106.	6; Poor
Vinther et al. (2017)	Denmark	Mixed methods	Interviews: Nine participants (6 staff members, 1 nursing home manager, 1 representative for the municipality, 1 local risk manager) Workshop and observed a staff meeting: Number of participants or number of observations was not specified.	Not reported	One nursing home	4; Poor
Vlaeyen et al. (2021)	Belgium	Quantitative	420 participants (420 residents) Nursing homes were asked to share the results of their existing internal fall registration.	658 falls	15 nursing homes, six were in urban locations, nine were suburban. There was an average of 83 beds. Nine were private non‐profit, five were public owned and 1 was private and for profit	11; Good
Wabe, Seaman, et al. (2022)	Australia	Quantitative	Deidentified clinical and care management data from the electronic software. Incident reports were obtained from the incident database.	27,878 incidents	25 residential aged‐care facilities	9; Fair
Wabe, Siette, et al. (2022)	Australia	Quantitative	Falls were reported using a standardized incident form.	27,696 incidents	25 residential aged‐care facilities	10; Fair
Wagner et al. (2005)	Canada	Quantitative	910 participants (910 residents)	426 fall incidents reported	Six for profit nursing homes. The number of beds in the nursing homes ranged from 120 to 186. Five of the homes were part of a chain.	12; Good
Wagner et al. (2008)	Canada	Quantitative	Intervention: 104 participants Control: 103 participants Three nursing homes used a menu‐driven incident reporting system while another three nurses continued using their existing incident report to document falls.	Not reported	Six for profit nursing homes. The number of beds in the nursing homes ranged from 120 to 186. Five of the homes were part of a corporation, and one was a free‐standing facility. Four of the homes were in an urban setting.	12; Good
Wagner et al. (2010)	Canada	Qualitative	41 participants (20 registered nurses/registered practical nurses and 21 personal support workers)	Not reported.	Four long‐term care facilities that provide intermediate‐level nursing care.	11; Good
Wagner et al. (2012)	Canada	Quantitative	1180 participants (582 registered nurses, 592 registered practical nurses, 6 people not accounted for in paper)	Not reported	Not reported	9; Fair
Wagner, Castle, and Handler (2013)	USA	Quantitative	399 participants (399 nursing home administrators)	Not reported	399 nursing homes. 60.4% were NHs in the 41–99 bed range, with only 4% greater than 200 beds. Profit status was evenly distributed between for profit (41.1%) and non‐profit (42.1%) homes.	10; Fair
Wagner, Castle, Reid, and Stone (2013)	USA	Mixed methods	32 participants (32 state department of health representatives)	Not reported	Not reported	7; Poor
Wagner, Damianakis, Pho, and Tourangeau (2013)	Canada	Mixed methods	245 participants (breakdown of registered nurses and registered practical nurses not reported)	Not reported	Not reported	8; Poor
White (2001)	Australia	Mixed methods	111 participants (breakdown of registered nurses and registered practical nurses not reported)	Not reported	104 high care residential aged‐care facilities.	10; Fair
Whitney et al. (2017)	UK	Quantitative	191 participants (191 residents) Falls were measured during the intervention period and 6‐month after using incident reports	Intervention: 6‐month follow‐up = 78 falls Control: 6‐month follow‐up = 41 falls	Nine care homes with 400 residents in total.	14; Very good
Wilson et al. (2011)	Australia	Quantitative	602 participants (602 residents) Fall data was obtained by reviewing nursing notes and incident reports	Not reported	51 residential aged‐care facilities	10; Fair
Yang et al. (2015)	Canada	Quantitative	Reviewed fall video with a validated questionnaire compared with information recorded on fall incident reports.	863 incidents of falls	Two long‐term care facilities. One facility has 312 beds which is for profit, the second has 236 beds and was not‐for‐profit.	8; Poor

### 3.2 Safety incident reporting systems and processes

All frequency data for safety incident reporting systems and processes are reported in Table 2.

**TABLE 2:** Frequency data on safety incident reporting systems and processes used in care homes.

Mechanism for reporting safety incidents	Number of studies (references)
Reporting all incidents	25 (Anderson et al., 2011; Barker et al., 2009; Carroll‐Solomon & Denny, 2005; Crespin et al., 2010; Desai et al., 2011; Desai, Williams, Greene, Pierson, Caprio, & Hansen, 2013a, 2013b; Desai, Williams, Greene, Pierson, & Hansen, 2013; Gibson et al., 2008; Greene et al., 2005; Hansen et al., 2010; Johnson & Madan, 2017; Joyce, 2020; Lane, 2009; Lane et al., 2014; Lundström et al., 2007; McGrane et al., 2022; Milligan et al., 2011; Pierson et al., 2007; Prang & Jelsness‐Jorgensen, 2014; Sluggett, Chen, et al., 2020; Storli et al., 2016; Theodos, 2004; Vinther et al., 2017; Yang et al., 2015)
Mandatory reporting	9 (Dugré et al., 2021; Flynn et al., 2002; Fuller et al., 2022; Handler et al., 2004; Hrib et al., 2013; Milligan et al., 2014; Myhre, Saga, et al., 2020; Rask et al., 2007; Wagner et al., 2012)
Duplicating system	8 (Greene et al., 2010; Hansen et al., 2006; Millet, 2013; Serre et al., 2022; Shmueli et al., 2014; Tariq et al., 2012; Wagner et al., 2005, 2008)
Voluntary reporting	3 (Baril et al., 2014; Blanchard et al., 2022; Liukka et al., 2021)
Discussion of errors with colleague/manager	2 (Myhre, Malmedal, et al., 2020; Wagner et al., 2012)
Formulation of intervention	1 (Kobayashi & Sugai, 2006)

Method used to facilitate safety incident reporting	Number of studies (references)
Electronic computerized reporting	40 (Blanchard et al., 2022; Carroll‐Solomon & Denny, 2005; Crespin et al., 2010; Desai et al., 2011; Desai, Williams, Greene, Pierson, Caprio, & Hansen, 2013a, 2013b; Desai, Williams, Greene, Pierson, & Hansen, 2013; Dugré et al., 2021; Francis‐Coad et al., 2018; Goh et al., 2021; Greene et al., 2005, 2011; Hansen et al., 2006, 2010; Jogerst et al., 2008; Joyce, 2020; Lane, 2009; Lane et al., 2014; Liukka et al., 2021; Mak et al., 2022; McDerby et al., 2019; Milligan et al., 2011, 2014; Myhre, Saga, et al., 2020; Pierson et al., 2007; Prang & Jelsness‐Jorgensen, 2014; Rahim‐Jamal et al., 2008; Rask et al., 2007; Sjogren et al., 2015; Sluggett, Chen, et al., 2020; Sluggett, Hopkins, et al., 2020; St Clair et al., 2021; Tommasini et al., 2008; Toots et al., 2019; Verrue et al., 2011; Vinther et al., 2017; Wagner et al., 2008, 2012; Wagner, Castle, Reid, & Stone, 2013)
Paper‐based/typed form	4 (Al‐Oraibi et al., 2012; Fuller et al., 2022; McCloskey et al., 2017; Schuengel et al., 2020)
Hybrid—Hand written then typed/imported	4 (Greene et al., 2010; Myhre, Malmedal, et al., 2020; Tariq et al., 2012; Wagner et al., 2005)
Mixed—Some facilities used paper‐based, some used electronic	4 (Gaskin et al., 2012; Hrib et al., 2013; Millet, 2013; Wagner, Castle, & Handler, 2013)
Hybrid—Verbal then written	1 (Wagner et al., 2010)

People reporting safety incidents	Number of studies (references)
Specific healthcare professionals' roles: nursing staff	15 (Anderson et al., 2011; Baril et al., 2014; Fuller et al., 2022; Handler et al., 2004; Jogerst et al., 2008; McCloskey et al., 2017; Myhre, Saga, et al., 2020; Prang & Jelsness‐Jorgensen, 2014; Rask et al., 2007; Shmueli et al., 2014; Storli et al., 2016; Theodos, 2004; Wagner et al., 2005, 2008; Yang et al., 2015)
Generic staff categories	11 (Al‐Oraibi et al., 2012; Barker et al., 2009; Blanchard et al., 2022; Brett et al., 2019; Dugré et al., 2021; Joyce, 2020; Kosse et al., 2015; Pierson et al., 2007; Robinovitch et al., 2013; Schuengel et al., 2020; Sluggett, Hopkins, et al., 2020)
Unspecific healthcare professionals' roles	8 (Arain et al., 2016; Desai, Williams, Greene, Pierson, Caprio, & Hansen, 2013a, 2013b; Desai, Williams, Greene, Pierson, & Hansen, 2013; Liukka et al., 2021; Milligan, 2012; Milligan et al., 2011, 2014)
Mix—Clinical and non‐clinical staff	8 (Greene et al., 2005, 2010; Hewitt et al., 2018; Hrib et al., 2013; Kobayashi & Sugai, 2006; Lundström et al., 2007; Millet, 2013; Tariq et al., 2012)
Non‐clinician nursing home staff such as carers/nursing aids	4 (McCloskey et al., 2017; Myhre, Malmedal, et al., 2020; Tommasini et al., 2008; Vinther et al., 2017)
All staff	3 (Carroll‐Solomon & Denny, 2005; Hansen et al., 2010; Johnson & Madan, 2017)
Mix—Clinical staff, non‐clinical staff, relatives or residents	2 (Myhre, Malmedal, et al., 2020; Vinther et al., 2017)
Mix—Clinical and pharmacy staff	1 (Flynn et al., 2002)
Specific healthcare professionals' roles: physician	1 (Sjogren et al., 2015)

Use of safety incident report data	Number of studies (references)
Review and analyse incident reports to summarize data/identify trends (e.g. errors made repeatedly)	15 (Carroll‐Solomon & Denny, 2005; Crespin et al., 2010; Greene et al., 2005, 2011; Hansen et al., 2010; Johnson & Madan, 2017; Pierson et al., 2007; Rask et al., 2007; Schuengel et al., 2020; Theodos, 2004; Verrue et al., 2011; Wagner et al., 2005, 2008; Wagner, Castle, & Handler, 2013; Wagner, Castle, Reid, & Stone, 2013)
Information shared with staff and internal departments (e.g. quality assurance)	9 (Greene et al., 2011; Handler et al., 2004; Hughes & Lapane, 2006; Myhre, Saga, et al., 2020; Schuengel et al., 2020; Tariq et al., 2012; Theodos, 2004; Vinther et al., 2017; Wagner et al., 2012)
Analyse incident reports for organizational learning (e.g. change policy) or to make workplace changes (e.g. implement training)	7 (Goh et al., 2021; Greene et al., 2011; Handler et al., 2004; Liukka et al., 2021; Millet, 2013; Serre et al., 2022; Vinther et al., 2017)
Incidents reported to professional external organizations	6 (Daly & Jogerst, 2005; Johnson & Madan, 2017; Myhre, Malmedal, et al., 2020; Sjogren et al., 2015; Vinther et al., 2017; Wagner, Castle, & Handler, 2013)
Lack of feedback/dissemination of feedback from reports	5 (Andersson & Hjelm, 2017; Handler et al., 2004; Myhre, Malmedal, et al., 2020; Prang & Jelsness‐Jorgensen, 2014; Wagner, Damianakis, Pho, & Tourangeau, 2013)
Incidents reported to family/guardian	3 (Serre et al., 2022; Wagner et al., 2010, 2012)
Information used to generate visual outputs (e.g. graphs and charts)	2 (Greene et al., 2011; Wagner et al., 2005)
Unclear what feedback practices exist	1 (Storli et al., 2016)

#### 3.2.1 Process of reporting safety incidents

Forty‐eight papers documented some information related to how safety incidents are reported, with nine studies specifically reporting that incident reporting was mandatory (Dugré et al., 2021; Flynn et al., 2002; Fuller et al., 2022; Handler et al., 2004; Hrib et al., 2013; Milligan et al., 2014; Myhre, Saga, et al., 2020; Rask et al., 2007; Wagner et al., 2012), and three reported incident reporting being voluntary (Baril et al., 2014; Blanchard et al., 2022; Liukka et al., 2021). Out of the papers that reported data under this category, 25 highlighted that they encouraged staff to report all incidents that occurred (Anderson et al., 2011; Barker et al., 2009; Carroll‐Solomon & Denny, 2005; Crespin et al., 2010; Desai et al., 2011; Desai, Williams, Greene, Pierson, Caprio, & Hansen, 2013a, 2013b; Desai, Williams, Greene, Pierson, & Hansen, 2013; Gibson et al., 2008; Greene et al., 2005; Hansen et al., 2010; Johnson & Madan, 2017; Joyce, 2020; Lane, 2009; Lane et al., 2014; Lundström et al., 2007; McGrane et al., 2022; Milligan et al., 2011; Pierson et al., 2007; Prang & Jelsness‐Jorgensen, 2014; Sluggett, Chen, et al., 2020; Storli et al., 2016; Theodos, 2004; Vinther et al., 2017; Yang et al., 2015).

#### 3.2.2 Method(s) used to report safety incidents

The methods used to report incidents indicate that the most used method is through a formal, electronic computerized reporting system (*n* = 40) (Blanchard et al., 2022; Carroll‐Solomon & Denny, 2005; Crespin et al., 2010; Desai et al., 2011; Desai, Williams, Greene, Pierson, Caprio, & Hansen, 2013a, 2013b; Desai, Williams, Greene, Pierson, & Hansen, 2013; Dugré et al., 2021; Francis‐Coad et al., 2018; Goh et al., 2021; Greene et al., 2005, 2011; Hansen et al., 2006, 2010; Jogerst et al., 2008; Joyce, 2020; Lane, 2009; Lane et al., 2014; Liukka et al., 2021; Mak et al., 2022; McDerby et al., 2019; Milligan et al., 2011, 2014; Myhre, Saga, et al., 2020; Pierson et al., 2007; Prang & Jelsness‐Jorgensen, 2014; Rahim‐Jamal et al., 2008; Rask et al., 2007; Sjogren et al., 2015; Sluggett, Chen, et al., 2020; Sluggett, Hopkins, et al., 2020; St Clair et al., 2021; Tommasini et al., 2008; Toots et al., 2019; Verrue et al., 2011; Vinther et al., 2017; Wagner et al., 2008, 2012; Wagner, Castle, Reid, & Stone, 2013), where incidents are reported directly onto dedicated software or web‐based system using a computer or hand‐held device. The specific method used to report incidents was not clear in a large proportion of papers where it was documented that incident reports were reviewed or obtained as part of the study, but not specifically documenting if these were handwritten forms, forms completed on an electronic device, or on an electronic system. Few papers (*n* = 4) (Al‐Oraibi et al., 2012; Fuller et al., 2022; McCloskey et al., 2017; Schuengel et al., 2020) specifically reported using a purely handwritten form, which was typically in the form of a paper‐based template or form, rather than free‐hand (unstructured) reporting.

#### 3.2.3 People reporting safety incidents

Most studies did not report who completed safety incident reports. Of those who specifically documented who reported incidents within nursing or care homes, the most referenced were nursing staff (*n* = 15) (Anderson et al., 2011; Baril et al., 2014; Fuller et al., 2022; Handler et al., 2004; Jogerst et al., 2008; McCloskey et al., 2017; Myhre, Saga, et al., 2020; Prang & Jelsness‐Jorgensen, 2014; Rask et al., 2007; Shmueli et al., 2014; Storli et al., 2016; Theodos, 2004; Wagner et al., 2005, 2008; Yang et al., 2015). However, a broad generic category was developed given that papers (*n* = 11) were often unclear about who had reported (Al‐Oraibi et al., 2012; Barker et al., 2009; Blanchard et al., 2022; Brett et al., 2019; Dugré et al., 2021; Joyce, 2020; Kosse et al., 2015; Pierson et al., 2007; Robinovitch et al., 2013; Schuengel et al., 2020; Sluggett, Hopkins, et al., 2020).

#### 3.2.4 Use of safety incident report data

Several of the included studies stressed that safety incident reports enabled the organization to review the incidents, often to generate summary reports and/or identify trends across incidents (*n* = 15) (Carroll‐Solomon & Denny, 2005; Crespin et al., 2010; Greene et al., 2005, 2011; Hansen et al., 2010; Johnson & Madan, 2017; Pierson et al., 2007; Rask et al., 2007; Schuengel et al., 2020; Theodos, 2004; Verrue et al., 2011; Wagner et al., 2005, 2008; Wagner, Castle, & Handler, 2013; Wagner, Castle, Reid, & Stone, 2013). Two of the papers specifically documented generating visual outputs such as graphs as part of the review process (Greene et al., 2011; Wagner et al., 2005). It was also highlighted that safety incident reports were often reviewed for the purpose of organizational learning or to implement changes to improve resident care (*n* = 7) (Goh et al., 2021; Greene et al., 2011; Handler et al., 2004; Liukka et al., 2021; Millet, 2013; Serre et al., 2022; Vinther et al., 2017), such as changing policies or implementing staff training. On some occasions, the incidents were shared with external organization (*n* = 6) (Daly & Jogerst, 2005; Johnson & Madan, 2017; Myhre, Malmedal, et al., 2020; Sjogren et al., 2015; Vinther et al., 2017; Wagner, Castle, & Handler, 2013), but more often with staff internal to the home and/or organization (*n* = 9) (Greene et al., 2011; Handler et al., 2004; Hughes & Lapane, 2006; Myhre, Saga, et al., 2020; Schuengel et al., 2020; Tariq et al., 2012; Theodos, 2004; Vinther et al., 2017; Wagner et al., 2012). Three studies specifically documented informing family or guardians of the incident. It was noted in multiple papers that there was a lack of feedback from the reporters of the incident (*n* = 5) (Andersson & Hjelm, 2017; Handler et al., 2004; Myhre, Malmedal, et al., 2020; Prang & Jelsness‐Jorgensen, 2014; Wagner, Damianakis, Pho, & Tourangeau, 2013).

#### 3.2.5 Information captured by incident reporting systems

**FIGURE 3:**
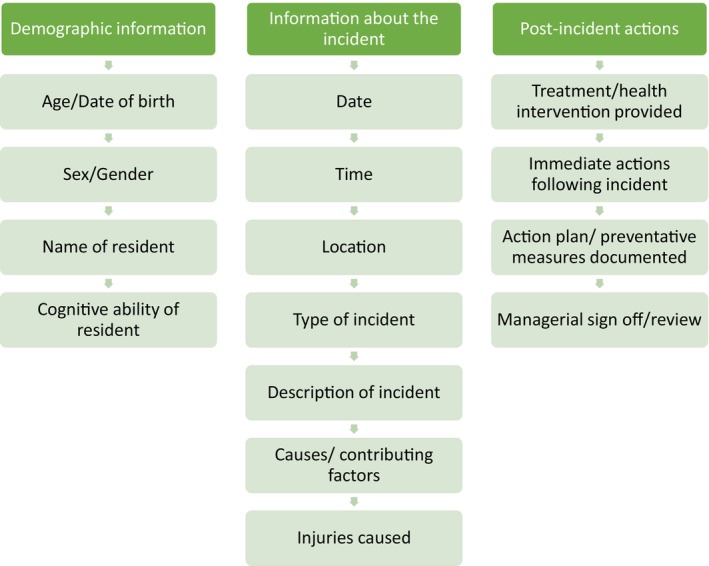
Summary of incident report data captured by incident reporting systems in care homes.

Thirty‐five papers reported data on the information captured by incident reporting systems (see Figure 3 for a summary and Table 3 for frequency data). These data were grouped into three categories: demographic information about the resident, information about the incident and post‐incident actions. A smaller number of papers reported on either falls‐specific information or medication‐specific information, suggesting that these incident types require more bespoke reporting (see Table 3). Frequently obtained information about the resident included the resident age (*n* = 11) (Al‐Oraibi et al., 2012; Blanchard et al., 2022; Carroll‐Solomon & Denny, 2005; Desai et al., 2011; Desai, Williams, Greene, Pierson, Caprio, & Hansen, 2013a, 2013b; Greene et al., 2010; Hansen et al., 2010; Johnson & Madan, 2017; Pierson et al., 2007; Wabe, Siette, et al., 2022), sex or gender (*n* = 7) (Al‐Oraibi et al., 2012; Carroll‐Solomon & Denny, 2005; Desai et al., 2011; Desai, Williams, Greene, Pierson, Caprio, & Hansen, 2013a, 2013b; Pierson et al., 2007; Wabe, Siette, et al., 2022), name (*n* = 6) (Carroll‐Solomon & Denny, 2005; Fuller et al., 2022; Johnson & Madan, 2017; Tariq et al., 2012; Theodos, 2004; Wagner et al., 2005), and the cognitive ability of the resident (*n* = 5) (Desai et al., 2011; Desai, Williams, Greene, Pierson, Caprio, & Hansen, 2013a, 2013b; Hansen et al., 2010; Pierson et al., 2007). In relation to the incident itself, commonly captured incident information consisted of the time (*n* = 19) (Al‐Oraibi et al., 2012; Blanchard et al., 2022; Desai et al., 2011; Dugré et al., 2021; Fuller et al., 2022; Gibson et al., 2008; Greene et al., 2010; Johnson & Madan, 2017; Kobayashi & Sugai, 2006; Kosse et al., 2015; Millet, 2013; Rask et al., 2007; Toots et al., 2019; Wabe, Seaman, et al., 2022; Wabe, Siette, et al., 2022; Wagner et al., 2005, 2008, 2010; Yang et al., 2015), the date (*n* = 16) (Baril et al., 2014; Blanchard et al., 2022; Desai et al., 2011; Dugré et al., 2021; Fuller et al., 2022; Gibson et al., 2008; Hansen et al., 2010; Johnson & Madan, 2017; Millet, 2013; Pierson et al., 2007; St Clair et al., 2021; Tariq et al., 2012; Theodos, 2004; Wabe, Seaman, et al., 2022; Wabe, Siette, et al., 2022; Wagner et al., 2005), the location the incident occurred (*n* = 13) (Al‐Oraibi et al., 2012; Dugré et al., 2021; Johnson & Madan, 2017; Kobayashi & Sugai, 2006; Kosse et al., 2015; Millet, 2013; Rask et al., 2007; Schuengel et al., 2020; Toots et al., 2019; Wabe, Seaman, et al., 2022; Wabe, Siette, et al., 2022; Wagner et al., 2008; Yang et al., 2015), the specific type of incident that occurred (*n* = 12) (Carroll‐Solomon & Denny, 2005; Crespin et al., 2010; Desai et al., 2011; Desai, Williams, Greene, Pierson, Caprio, & Hansen, 2013a, 2013b; Desai, Williams, Greene, Pierson, & Hansen, 2013; Fuller et al., 2022; Greene et al., 2010; Millet, 2013; Schuengel et al., 2020; Wabe, Seaman, et al., 2022), often with an description of the incident (*n* = 10) (Blanchard et al., 2022; Brett et al., 2019; Carroll‐Solomon & Denny, 2005; Dugré et al., 2021; Fuller et al., 2022; Johnson & Madan, 2017; McGrane et al., 2022; Millet, 2013; St Clair et al., 2021; Tariq et al., 2012), possible causes or contributing factors that led to the incident (*n* = 12) (Baril et al., 2014; Carroll‐Solomon & Denny, 2005; Crespin et al., 2010; Desai et al., 2011; Desai, Williams, Greene, Pierson, Caprio, & Hansen, 2013a, 2013b; Greene et al., 2010; Hansen et al., 2010; Millet, 2013; Rask et al., 2007; Shmueli et al., 2014; St Clair et al., 2021; Wagner et al., 2005, 2008; Yang et al., 2015) and any injuries or consequences caused to the person involved in the incident (*n* = 25) (Al‐Oraibi et al., 2012; Baril et al., 2014; Carroll‐Solomon & Denny, 2005; Crespin et al., 2010; Desai et al., 2011; Fuller et al., 2022; Gibson et al., 2008; Greene et al., 2010; Hansen et al., 2010; Johnson & Madan, 2017; Kobayashi & Sugai, 2006; Kosse et al., 2015; Lane, 2009; McGrane et al., 2022; Pierson et al., 2007; Rask et al., 2007; Schuengel et al., 2020; St Clair et al., 2021; Theodos, 2004; Toots et al., 2019; Wabe, Seaman, et al., 2022; Wabe, Siette, et al., 2022; Wagner et al., 2005, 2008; Yang et al., 2015).

**TABLE 3:** Frequency data on information captured in safety incident reports.

Description of resident/home	Number of studies (references)
Age/date of birth	11 (Al‐Oraibi et al., 2012; Blanchard et al., 2022; Carroll‐Solomon & Denny, 2005; Desai et al., 2011; Desai, Williams, Greene, Pierson, Caprio, & Hansen, 2013a, 2013b; Greene et al., 2010; Hansen et al., 2010; Johnson & Madan, 2017; Pierson et al., 2007; Wabe, Siette, et al., 2022)
Sex or gender	7 (Al‐Oraibi et al., 2012; Carroll‐Solomon & Denny, 2005; Desai et al., 2011; Desai, Williams, Greene, Pierson, Caprio, & Hansen, 2013a, 2013b; Pierson et al., 2007; Wabe, Siette, et al., 2022)
Name	6 (Carroll‐Solomon & Denny, 2005; Fuller et al., 2022; Johnson & Madan, 2017; Tariq et al., 2012; Theodos, 2004; Wagner et al., 2005)
Cognitive ability	5 (Desai et al., 2011; Desai, Williams, Greene, Pierson, Caprio, & Hansen, 2013a, 2013b; Hansen et al., 2010; Pierson et al., 2007)
Details relating to reporter (name/initials/job)	4 (Blanchard et al., 2022; Dugré et al., 2021; Millet, 2013; Wagner et al., 2005)
Room number	3 (Carroll‐Solomon & Denny, 2005; Fuller et al., 2022; Johnson & Madan, 2017)
Facility	2 (Fuller et al., 2022; Tariq et al., 2012)
Resident unit	2 (Tariq et al., 2012; Wagner et al., 2005)
Ability to direct own care	1 (Greene et al., 2010)
Country of birth	1 (Wabe, Siette, et al., 2022)
Diagnosis prior to incident	1 (Johnson & Madan, 2017)
Date of admission	1 (Theodos, 2004)
Shift	1 (Pierson et al., 2007)

Information about the incident	Number of studies (references)
Injuries/consequences caused	25 (Al‐Oraibi et al., 2012; Baril et al., 2014; Carroll‐Solomon & Denny, 2005; Crespin et al., 2010; Desai et al., 2011; Fuller et al., 2022; Gibson et al., 2008; Greene et al., 2010; Hansen et al., 2010; Johnson & Madan, 2017; Kobayashi & Sugai, 2006; Kosse et al., 2015; Lane, 2009; McGrane et al., 2022; Pierson et al., 2007; Rask et al., 2007; Schuengel et al., 2020; St Clair et al., 2021; Theodos, 2004; Toots et al., 2019; Wabe, Seaman, et al., 2022; Wabe, Siette, et al., 2022; Wagner et al., 2005, 2008; Yang et al., 2015)
Time of incident	19 (Al‐Oraibi et al., 2012; Blanchard et al., 2022; Desai et al., 2011; Dugré et al., 2021; Fuller et al., 2022; Gibson et al., 2008; Greene et al., 2010; Johnson & Madan, 2017; Kobayashi & Sugai, 2006; Kosse et al., 2015; Millet, 2013; Rask et al., 2007; Toots et al., 2019; Wabe, Seaman, et al., 2022; Wabe, Siette, et al., 2022; Wagner et al., 2005, 2008, 2010; Yang et al., 2015)
Date of incident	16 (Baril et al., 2014; Blanchard et al., 2022; Desai et al., 2011; Dugré et al., 2021; Fuller et al., 2022; Gibson et al., 2008; Hansen et al., 2010; Johnson & Madan, 2017; Millet, 2013; Pierson et al., 2007; St Clair et al., 2021; Tariq et al., 2012; Theodos, 2004; Wabe, Seaman, et al., 2022; Wabe, Siette, et al., 2022; Wagner et al., 2005)
Location of incident	13 (Al‐Oraibi et al., 2012; Dugré et al., 2021; Johnson & Madan, 2017; Kobayashi & Sugai, 2006; Kosse et al., 2015; Millet, 2013; Rask et al., 2007; Schuengel et al., 2020; Toots et al., 2019; Wabe, Seaman, et al., 2022; Wabe, Siette, et al., 2022; Wagner et al., 2008; Yang et al., 2015)
Possible causes/contributing factors of incident	12 (Baril et al., 2014; Carroll‐Solomon & Denny, 2005; Crespin et al., 2010; Desai et al., 2011; Desai, Williams, Greene, Pierson, Caprio, & Hansen, 2013a, 2013b; Greene et al., 2010; Hansen et al., 2010; Millet, 2013; Rask et al., 2007; Shmueli et al., 2014; St Clair et al., 2021; Wagner et al., 2005, 2008; Yang et al., 2015)
Type of incident/error	12 (Carroll‐Solomon & Denny, 2005; Crespin et al., 2010; Desai et al., 2011; Desai, Williams, Greene, Pierson, Caprio, & Hansen, 2013a, 2013b; Desai, Williams, Greene, Pierson, & Hansen, 2013; Fuller et al., 2022; Greene et al., 2010; Millet, 2013; Schuengel et al., 2020; Wabe, Seaman, et al., 2022)
Description of incident	10 (Blanchard et al., 2022; Brett et al., 2019; Carroll‐Solomon & Denny, 2005; Dugré et al., 2021; Fuller et al., 2022; Johnson & Madan, 2017; McGrane et al., 2022; Millet, 2013; St Clair et al., 2021; Tariq et al., 2012)
Personnel involved in incident	10 (Crespin et al., 2010; Desai et al., 2011; Desai, Williams, Greene, Pierson, Caprio, & Hansen, 2013a, 2013b; Desai, Williams, Greene, Pierson, & Hansen, 2013; Fuller et al., 2022; Greene et al., 2010; Hansen et al., 2010; Lane, 2009; Lane et al., 2014; Pierson et al., 2007)
Whether the incident was a repeat incident	6 (Crespin et al., 2010; Desai et al., 2011; Desai, Williams, Greene, Pierson, Caprio, & Hansen, 2013a, 2013b; Desai, Williams, Greene, Pierson, & Hansen, 2013; Pierson et al., 2007)
Did the incident occur within 7 days of transitioning into the home/was the resident moving into the facility	5 (Desai et al., 2011; Desai, Williams, Greene, Pierson, Caprio, & Hansen, 2013a, 2013b; Desai, Williams, Greene, Pierson, & Hansen, 2013; Pierson et al., 2007)
Severity of the incident/level of harm	5 (Al‐Oraibi et al., 2012; Baril et al., 2014; Kobayashi & Sugai, 2006; Pierson et al., 2007; Shmueli et al., 2014)
Details of person present at time of error/witnesses	3 (Johnson & Madan, 2017; Schuengel et al., 2020; Tariq et al., 2012)
Whether incident could have been prevented	1 (Johnson & Madan, 2017)
Day of the week	1 (Rask et al., 2007)

Additional medication‐specific incident information	Number of studies (references)
Medication error type (including wrong patient, wrong dose, wrong drug, wrong administration, wrong follow‐up or other)	9 (Desai et al., 2011; Desai, Williams, Greene, Pierson, Caprio, & Hansen, 2013a, 2013b; Desai, Williams, Greene, Pierson, & Hansen, 2013; Fuller et al., 2022; Hansen et al., 2010; Lane, 2009; Lane et al., 2014; Pierson et al., 2007)
Phase of medication process (including prescribing, dispensing, documenting, administrating, or monitoring)	9 (Crespin et al., 2010; Desai et al., 2011; Desai, Williams, Greene, Pierson, Caprio, & Hansen, 2013a, 2013b; Desai, Williams, Greene, Pierson, & Hansen, 2013; Greene et al., 2010; Hansen et al., 2010; Lane et al., 2014; Pierson et al., 2007)
Specific medication error cause (including product issues, recording issues, dispensing issues, facility issues, personnel issues, or other)	7 (Crespin et al., 2010; Desai et al., 2011; Desai, Williams, Greene, Pierson, Caprio, & Hansen, 2013a; Hansen et al., 2010; Lane, 2009; Lane et al., 2014; Pierson et al., 2007)
Medication(s) involved	7 (Crespin et al., 2010; Desai et al., 2011; Desai, Williams, Greene, Pierson, Caprio, & Hansen, 2013a, 2013b; Greene et al., 2010; Hansen et al., 2010; Pierson et al., 2007)
Comments from pharmacist	1 (Tariq et al., 2012)
Number of medications	1 (Pierson et al., 2007)

Additional falls‐specific incident information	Number of studies (references)
Fall type/activity at time of fall (during transfer, during transport, from bed, found on floor, from chair or wheel‐chair, from exam table or equipment, when ambulating, when toileting, other)	4 (Carroll‐Solomon & Denny, 2005; Theodos, 2004; Wagner et al., 2008; Yang et al., 2015)
Witnessed fall, unwitnessed fall or near fall	4 (Carroll‐Solomon & Denny, 2005; Kosse et al., 2015; Wagner et al., 2005; Yang et al., 2015)
Known cause of imbalance/fall	3 (Theodos, 2004; Wagner et al., 2005; Yang et al., 2015)
History of falls	2 (Theodos, 2004; Wabe, Siette, et al., 2022)
Whether footwear was warn	2 (Wagner et al., 2005, 2008)
Place fall witnessed or incident found	1 (Kobayashi & Sugai, 2006)
Fall protocol in place	1 (Carroll‐Solomon & Denny, 2005)
Are mobility aids used	1 (Yang et al., 2015)
Whether staff were present during activity	1 (Wagner et al., 2005)
Risk of falls	1 (Wabe, Siette, et al., 2022)

Post‐incident actions	Number of studies (references)
Treatment/health interventions provided post‐incident (e.g. first aid, or transferred to hospital)	11 (Al‐Oraibi et al., 2012; Dugré et al., 2021; Gibson et al., 2008; Johnson & Madan, 2017; Kobayashi & Sugai, 2006; McGrane et al., 2022; St Clair et al., 2021; Tariq et al., 2012; Theodos, 2004; Wabe, Seaman, et al., 2022; Wabe, Siette, et al., 2022)
Immediate response/actions taken post incident	6 (Al‐Oraibi et al., 2012; Blanchard et al., 2022; Dugré et al., 2021; Fuller et al., 2022; McGrane et al., 2022; Tariq et al., 2012)
Action plan moving forward/Preventive measures implemented	5 (Dugré et al., 2021; Johnson & Madan, 2017; Kobayashi & Sugai, 2006; McGrane et al., 2022; Wagner et al., 2008)
Signature or review from nurse or manager	4 (Fuller et al., 2022; Millet, 2013; Tariq et al., 2012; Theodos, 2004)
Who had been informed about the incident (including family, guardian, doctors)	4 (Dugré et al., 2021; Johnson & Madan, 2017; McGrane et al., 2022; Tariq et al., 2012)
Outcomes of investigations	3 (Dugré et al., 2021; Tariq et al., 2012; Theodos, 2004)
Follow‐up conducted from staff	2 (Fuller et al., 2022; Schuengel et al., 2020)
Date of investigations	1 (Theodos, 2004)

Post‐incident information included what treatment or health intervention was completed following the incident (such as first aid administered or transferring resident to hospital) (*n* = 11) (Al‐Oraibi et al., 2012; Dugré et al., 2021; Gibson et al., 2008; Johnson & Madan, 2017; Kobayashi & Sugai, 2006; McGrane et al., 2022; St Clair et al., 2021; Tariq et al., 2012; Theodos, 2004; Wabe, Seaman, et al., 2022; Wabe, Siette, et al., 2022), as well as other immediate actions completed following the incident (*n* = 6) (Al‐Oraibi et al., 2012; Blanchard et al., 2022; Dugré et al., 2021; Fuller et al., 2022; McGrane et al., 2022; Tariq et al., 2012), and specifically making others aware of the incident (such as calling GP, family members, guardians; *n* = 4) (Dugré et al., 2021; Johnson & Madan, 2017; McGrane et al., 2022; Tariq et al., 2012). Five papers documented data on actions and preventive measures that will be implemented following the incident, for future errors to be prevented (Dugré et al., 2021; Johnson & Madan, 2017; Kobayashi & Sugai, 2006; McGrane et al., 2022; Wagner et al., 2008). Lastly, signoff or review of the incident report by nurse or management was documented in few papers (*n* = 4) (Fuller et al., 2022; Millet, 2013; Tariq et al., 2012; Theodos, 2004).

### 3.3 Types of incidents

The following section presents the results of coding studies against the WHO International Classification for Patient Safety conceptual framework (Runciman et al., 2009; Sherman et al., 2009), firstly by the types of incidents captured by reporting systems (Table 4) and then other aspects of incidents including characteristics, detection, contributing and mitigating factors, ameliorating actions, actions taken to reduce risk and outcomes (Table 5).

**TABLE 4:** Frequency of studies reporting patient safety incident categories based on WHO conceptual framework for the international classification for patient safety (Runciman et al., 2009; Sherman et al., 2009).

Incident types	Number of studies	Incident types	Number of studies
Behaviour		Infection	
Staff		Type of organism	
Harassment	1 (Smith et al., 2022)	Bacteria	2 (Kapoor et al., 2019; Kepner et al., 2022)
Physical assault	1 (McCloskey et al., 2017)	Causative organism not identified	2 (Chen et al., 2022; Lee & Cho, 2022)
Patients		Virus	1 (Kepner et al., 2022)
Physical assault	10 (Blanchard et al., 2022; Gil & Capelas, 2022; Joyce, 2020; Kim et al., 2017; Lachs et al., 2016; Lundström et al., 2007; McCloskey et al., 2017; Shmueli et al., 2014; St Clair et al., 2021; Wagner, Castle, Reid, & Stone, 2013)	Type/site of infection	
Verbal aggression	7 (Blanchard et al., 2022; Gil & Capelas, 2022; Joyce, 2020; Kim et al., 2017; Lachs et al., 2016; Lundström et al., 2007; St Clair et al., 2021)	Pneumonia	1 (Kapoor et al., 2019)
Sexual assault	6 (Gil & Capelas, 2022; Joyce, 2020; Lachs et al., 2016; Lundström et al., 2007; Smith et al., 2022; Wagner, Castle, Reid, & Stone, 2013)	Soft tissue	1 (Chen et al., 2022)
Intended self‐harm/suicide	4 (Andersson & Hjelm, 2017; Fuller et al., 2022; Shmueli et al., 2014; Wagner, Castle, Reid, & Stone, 2013)		
		Medication/intravenous fluids	
Wandering/absconding	2 (Kim et al., 2017; St Clair et al., 2021)	Medication/fluid involved	
Harassment	1 (Smith et al., 2022)	Medication list	27 (Arain et al., 2016; Baril et al., 2014; Barker et al., 2002; Carroll‐Solomon & Denny, 2005; Crespin et al., 2010; Desai et al., 2011; Desai, Williams, Greene, Pierson, Caprio, & Hansen, 2013a, 2013b; Desai, Williams, Greene, Pierson, & Hansen, 2013; Dugré et al., 2021; Fuller et al., 2022; Gray‐Miceli et al., 2010; Greene et al., 2010; Gurwitz et al., 2005; Handler et al., 2004; Hansen et al., 2006, 2010; Lee & Cho, 2022; McDerby et al., 2019; Milligan, 2012; Milligan et al., 2011; Pierson et al., 2007; Rahim‐Jamal et al., 2008; Ray et al., 2002; Shmueli et al., 2014; Sluggett, Chen, et al., 2020; Sluggett, Hopkins, et al., 2020)
Noncompliant/uncooperative/obstructive	1 (Rask et al., 2007)	Use process	
Clinical administration		Administration	22 (Andersson & Hjelm, 2017; Arain et al., 2016; Baril et al., 2014; Barker et al., 2002; Crespin et al., 2010; Desai et al., 2011; Desai, Williams, Greene, Pierson, Caprio, & Hansen, 2013a, 2013b; Desai, Williams, Greene, Pierson, & Hansen, 2013; Dugré et al., 2021; Greene et al., 2005, 2010; Gurwitz et al., 2005; Handler et al., 2004; Lane, 2009; Lane et al., 2014; McDerby et al., 2019; Milligan, 2012; Milligan et al., 2011; Pierson et al., 2007; Shmueli et al., 2014)
Process		Prescribing	14 (Crespin et al., 2010; Desai et al., 2011; Desai, Williams, Greene, Pierson, Caprio, & Hansen, 2013a, 2013b; Desai, Williams, Greene, Pierson, & Hansen, 2013; Dugré et al., 2021; Greene et al., 2005, 2010; Hansen et al., 2006; Lane et al., 2014; Milligan, 2012; Pierson et al., 2007; Ray et al., 2002; Verrue et al., 2011)
Transfer of care	4 (Crespin et al., 2010; Desai et al., 2011; Desai, Williams, Greene, Pierson, & Hansen, 2013; Francis‐Coad et al., 2018)	Preparation/dispensing	14 (Baril et al., 2014; Crespin et al., 2010; Desai, Williams, Greene, Pierson, Caprio, & Hansen, 2013a, 2013b; Desai, Williams, Greene, Pierson, & Hansen, 2013; Fuller et al., 2022; Greene et al., 2010; Gurwitz et al., 2005; Handler et al., 2004; Hansen et al., 2010; Lane et al., 2014; McDerby et al., 2019; Milligan, 2012; Milligan et al., 2011)
Admission	2 (Lane, 2009; Lane et al., 2014)	Monitoring	14 (Crespin et al., 2010; Desai et al., 2011; Desai, Williams, Greene, Pierson, Caprio, & Hansen, 2013a, 2013b; Desai, Williams, Greene, Pierson, & Hansen, 2013; Greene et al., 2005, 2010, 2011; Gurwitz et al., 2005; Hansen et al., 2006; Lane, 2009; Lane et al., 2014; Milligan, 2012; Pierson et al., 2007)
Appointment	1 (Sjogren et al., 2015)	Delivery	10 (Andersson & Hjelm, 2017; Arain et al., 2016; Barker et al., 2002; Dugré et al., 2021; Flynn et al., 2002; Fuller et al., 2022; McDerby et al., 2019; Sluggett, Chen, et al., 2020; Sluggett, Hopkins, et al., 2020; Verrue et al., 2011)
Patient identification	1 (Sjogren et al., 2015)	Supply/ordering	7 (Desai et al., 2011; Greene et al., 2010, 2011; Gurwitz et al., 2005; Hansen et al., 2010; Milligan et al., 2011; Verrue et al., 2011)
Problem		Storage	2 (McDerby et al., 2019; Verrue et al., 2011)
Incomplete/inadequate	6 (Crespin et al., 2010; Desai et al., 2011; Desai, Williams, Greene, Pierson, & Hansen, 2013; Francis‐Coad et al., 2018; Lane, 2009; Lane et al., 2014)	Problem	
Clinical process/procedure		Wrong dose/strength of frequency	23 (Andersson & Hjelm, 2017; Arain et al., 2016; Baril et al., 2014; Barker et al., 2002; Crespin et al., 2010; Desai et al., 2011; Desai, Williams, Greene, Pierson, Caprio, & Hansen, 2013a, 2013b; Desai, Williams, Greene, Pierson, & Hansen, 2013; Dugré et al., 2021; Flynn et al., 2002; Fuller et al., 2022; Greene et al., 2005, 2010, 2011; Gurwitz et al., 2005; Handler et al., 2004; Lane, 2009; Lane et al., 2014; McDerby et al., 2019; Milligan, 2012; Milligan et al., 2011; Pierson et al., 2007)
Process		Wrong drug	19 (Arain et al., 2016; Barker et al., 2002; Crespin et al., 2010; Desai et al., 2011; Desai, Williams, Greene, Pierson, Caprio, & Hansen, 2013a, 2013b; Desai, Williams, Greene, Pierson, & Hansen, 2013; Dugré et al., 2021; Greene et al., 2005, 2010, 2011; Gurwitz et al., 2005; Handler et al., 2004; Hansen et al., 2010; Lane, 2009; McDerby et al., 2019; Milligan, 2012; Milligan et al., 2011; Pierson et al., 2007)
Procedure/treatment/intervention	28 (Arain et al., 2016; Baril et al., 2014; Barker et al., 2002; Capezuti et al., 2002, 2007; Crespin et al., 2010; Desai et al., 2011; Desai, Williams, Greene, Pierson, Caprio, & Hansen, 2013a, 2013b; Desai, Williams, Greene, Pierson, & Hansen, 2013; Flynn et al., 2002; Greene et al., 2005, 2010, 2011; Gurwitz et al., 2005; Handler et al., 2004; Hansen et al., 2006, 2010; Lane, 2009; Lane et al., 2014; McDerby et al., 2019; McGrane et al., 2022; Milligan, 2012; Milligan et al., 2011; Pierson et al., 2007; Rahim‐Jamal et al., 2008; Shmueli et al., 2014; Sluggett, Hopkins, et al., 2020)	Omitted medicine or dose	17 (Arain et al., 2016; Baril et al., 2014; Barker et al., 2002; Dugré et al., 2021; Flynn et al., 2002; Greene et al., 2005, 2010, 2011; Handler et al., 2004; Hansen et al., 2010; Lane, 2009; Lane et al., 2014; McDerby et al., 2019; Milligan, 2012; Milligan et al., 2011; Pierson et al., 2007; St Clair et al., 2021)
General care/management	7 (Al‐Oraibi et al., 2012; Blanchard et al., 2022; Carroll‐Solomon & Denny, 2005; Dugré et al., 2021; Fuller et al., 2022; Gil & Capelas, 2022; White, 2001)	Wrong patient	15 (Arain et al., 2016; Baril et al., 2014; Crespin et al., 2010; Desai et al., 2011; Desai, Williams, Greene, Pierson, Caprio, & Hansen, 2013a, 2013b; Desai, Williams, Greene, Pierson, & Hansen, 2013; Greene et al., 2005, 2010; Handler et al., 2004; Lane, 2009; Lane et al., 2014; Milligan, 2012; Milligan et al., 2011; Pierson et al., 2007)
Diagnosis/assessment	3 (Andersson & Hjelm, 2017; Kapoor et al., 2019; Sjogren et al., 2015)	Wrong route	13 (Baril et al., 2014; Barker et al., 2002; Crespin et al., 2010; Desai et al., 2011; Desai, Williams, Greene, Pierson, Caprio, & Hansen, 2013a, 2013b; Desai, Williams, Greene, Pierson, & Hansen, 2013; Flynn et al., 2002; Greene et al., 2005, 2010, 2011; Handler et al., 2004; Lane, 2009)
Problem		Wrong formulation or presentation	10 (Barker et al., 2002; Desai, Williams, Greene, Pierson, Caprio, & Hansen, 2013a; Flynn et al., 2002; Greene et al., 2005, 2010; Lane, 2009; McDerby et al., 2019; Milligan, 2012; Milligan et al., 2011; Verrue et al., 2011)
Incomplete/inadequate	29 (Andersson & Hjelm, 2017; Baril et al., 2014; Barker et al., 2002; Blanchard et al., 2022; Capezuti et al., 2002, 2007; Carroll‐Solomon & Denny, 2005; Crespin et al., 2010; Desai et al., 2011; Desai, Williams, Greene, Pierson, Caprio, & Hansen, 2013a; Dugré et al., 2021; Flynn et al., 2002; Fuller et al., 2022; Gil & Capelas, 2022; Greene et al., 2005, 2010, 2011; Gurwitz et al., 2005; Handler et al., 2004; Hansen et al., 2006, 2010; Lane, 2009; Lane et al., 2014; McGrane et al., 2022; Milligan, 2012; Milligan et al., 2011; Sjogren et al., 2015; White, 2001)	Adverse drug reaction	7 (Dugré et al., 2021; Gurwitz et al., 2005; Kapoor et al., 2019; Lane et al., 2014; Milligan, 2012; Milligan et al., 2011; Rahim‐Jamal et al., 2008)
Wrong patient	18 (Arain et al., 2016; Baril et al., 2014; Barker et al., 2002; Crespin et al., 2010; Desai et al., 2011; Desai, Williams, Greene, Pierson, Caprio, & Hansen, 2013a, 2013b; Desai, Williams, Greene, Pierson, & Hansen, 2013; Dugré et al., 2021; Flynn et al., 2002; Fuller et al., 2022; Greene et al., 2005, 2010; Handler et al., 2004; Lane, 2009; Lane et al., 2014; Milligan, 2012; Pierson et al., 2007)	Expired medicine	4 (Greene et al., 2005; Lane, 2009; Milligan, 2012; Milligan et al., 2011)
Not performed when indicated	15 (Baril et al., 2014; Barker et al., 2002; Crespin et al., 2010; Desai, Williams, Greene, Pierson, & Hansen, 2013; Dugré et al., 2021; Flynn et al., 2002; Gil & Capelas, 2022; Greene et al., 2005, 2010, 2011; Kapoor et al., 2019; Lane, 2009; Lane et al., 2014; McGrane et al., 2022; Pierson et al., 2007)	Wrong storage	3 (Arain et al., 2016; Milligan, 2012; Milligan et al., 2011)
Unavailable	5 (Fuller et al., 2022; Hansen et al., 2010; Milligan, 2012; Milligan et al., 2011; Pierson et al., 2007)	Wrong dispensing label/instruction	1 (Milligan et al., 2011)
Wrong process/service	1 (McDerby et al., 2019)	Wrong quantity	1 (Milligan, 2012)
Infrastructure		Medical device/equipment	
Involved		Type of property	
Fixture list	1 (O'Regan et al., 2022)	Device/equipment/property list	2 (Shmueli et al., 2014; Sjogren et al., 2015)
Documentation		Patient accidents	
Document involved		Falls	40 (Al‐Oraibi et al., 2012; Andersson & Hjelm, 2017; Arfken et al., 2001; Barker et al., 2009; Capezuti et al., 2002, 2007; Carroll‐Solomon & Denny, 2005; DeSure et al., 2013; Francis‐Coad et al., 2018; Gibson et al., 2008; Gray‐Miceli et al., 2010; Hewitt et al., 2018; Johnson & Madan, 2017; Joyce, 2020; Kapoor et al., 2019; Kobayashi & Sugai, 2006; Kosse et al., 2015; Lee & Cho, 2022; Lord et al., 2003; Mak et al., 2022; Mirolsky‐Scala & Kraemer, 2009; Neyens et al., 2009; Rask et al., 2007; Ray et al., 2002; Robinovitch et al., 2013; Shmueli et al., 2014; Sjogren et al., 2015; St Clair et al., 2021; Theodos, 2004; Tommasini et al., 2008; Toots et al., 2019; Vlaeyen et al., 2021; Wabe, Seaman, et al., 2022; Wabe, Siette, et al., 2022; Wagner et al., 2005, 2008; Wagner, Castle, Reid, & Stone, 2013; Whitney et al., 2017; Wilson et al., 2011; Yang et al., 2015)
Charts/medical records/assessments/consultations	16 (Andersson & Hjelm, 2017; Chen et al., 2022; Crespin et al., 2010; Dugré et al., 2021; Greene et al., 2005; Handler et al., 2004; Lane, 2009; McDerby et al., 2019; McGrane et al., 2022; Rahim‐Jamal et al., 2008; Rask et al., 2007; Sjogren et al., 2015; Wagner et al., 2005, 2008; White, 2001; Yang et al., 2015)	Type of fall	
Orders/requests	10 (Arain et al., 2016; Desai, Williams, Greene, Pierson, Caprio, & Hansen, 2013a, 2013b; Desai, Williams, Greene, Pierson, & Hansen, 2013; Greene et al., 2010, 2011; Hansen et al., 2006, 2010; Lane et al., 2014; St Clair et al., 2021)	Loss of balance	9 (Capezuti et al., 2002, 2007; Joyce, 2020; Lord et al., 2003; Mirolsky‐Scala & Kraemer, 2009; Robinovitch et al., 2013; Tommasini et al., 2008; Wagner et al., 2005; Yang et al., 2015)
Instructions/information/policies/procedures/guidelines	2 (Milligan, 2012; Milligan et al., 2011)	Slip	5 (Mirolsky‐Scala & Kraemer, 2009; Robinovitch et al., 2013; Tommasini et al., 2008; Wagner et al., 2005; Yang et al., 2015)
		Trip/stumble	4 (Mirolsky‐Scala & Kraemer, 2009; Robinovitch et al., 2013; Wagner et al., 2005; Yang et al., 2015)
Problem		Collapse	3 (Mirolsky‐Scala & Kraemer, 2009; Robinovitch et al., 2013; Yang et al., 2015)
Unclear/ambiguous/illegible/incomplete information in document	13 (Andersson & Hjelm, 2017; Crespin et al., 2010; Desai, Williams, Greene, Pierson, & Hansen, 2013; Greene et al., 2005; Handler et al., 2004; Hansen et al., 2010; Lane, 2009; Lane et al., 2014; McGrane et al., 2022; Rask et al., 2007; Wagner et al., 2005, 2008; Yang et al., 2015)	Fall involving (equipment)	
Document missing or unavailable	9 (Andersson & Hjelm, 2017; Chen et al., 2022; McDerby et al., 2019; McGrane et al., 2022; Milligan, 2012; Milligan et al., 2011; Rahim‐Jamal et al., 2008; Wagner et al., 2008; White, 2001)	Bed	9 (Capezuti et al., 2002, 2007; Carroll‐Solomon & Denny, 2005; Kobayashi & Sugai, 2006; Lord et al., 2003; Neyens et al., 2009; Shmueli et al., 2014; Theodos, 2004; Wagner et al., 2005)
Document for wrong patient or wrong document	2 (Greene et al., 2010, 2011)	Chair	5 (Carroll‐Solomon & Denny, 2005; Kobayashi & Sugai, 2006; Lord et al., 2003; Robinovitch et al., 2013; Theodos, 2004)
		Toilet	2 (Carroll‐Solomon & Denny, 2005; Kobayashi & Sugai, 2006)
		Therapeutic equipment	1 (Yang et al., 2015)

**TABLE 5:** Frequency of studies reporting categories of incident details, contributing factors and outcomes based on WHO conceptual framework for the international classification for patient safety (Runciman et al., 2009; Sherman et al., 2009).

Incident characteristics	Number of studies	Mitigating factors	Number of studies
People involved		Directed to patient	
Healthcare professional	33 (Andersson & Hjelm, 2017; Arain et al., 2016; Baril et al., 2014; Barker et al., 2002; Desai et al., 2011; Desai, Williams, Greene, Pierson, Caprio, & Hansen, 2013a; Desai, Williams, Greene, Pierson, & Hansen, 2013; Dugré et al., 2021; Flynn et al., 2002; Fuller et al., 2022; Gray‐Miceli et al., 2010; Greene et al., 2005, 2010, 2011; Gurwitz et al., 2005; Handler et al., 2004; Hansen et al., 2006, 2010; Johnson & Madan, 2017; Kobayashi & Sugai, 2006; Lane, 2009; Lane et al., 2014; McDerby et al., 2019; Mirolsky‐Scala & Kraemer, 2009; Pierson et al., 2007; Rahim‐Jamal et al., 2008; Sluggett, Chen, et al., 2020; Sluggett, Hopkins, et al., 2020; Toots et al., 2019; Verrue et al., 2011; Wagner et al., 2008; Wagner, Castle, & Handler, 2013a; White, 2001)	Management/treatment/care undertaken	9 (Al‐Oraibi et al., 2012; DeSure et al., 2013; Francis‐Coad et al., 2018; Gray‐Miceli et al., 2010; Joyce, 2020; Lundström et al., 2007; Mak et al., 2022; Neyens et al., 2009; Sluggett, Hopkins, et al., 2020)
Healthcare worker	10 (Blanchard et al., 2022; Gil & Capelas, 2022; Kosse et al., 2015; Lee & Cho, 2022; Lord et al., 2003; Lundström et al., 2007; McCloskey et al., 2017; McGrane et al., 2022; Smith et al., 2022; Wilson et al., 2011)	Patient referred	1 (McGrane et al., 2022)
Another patient	3 (Joyce, 2020; Lundström et al., 2007; Robinovitch et al., 2013)	Directed to staff	
Stage/phase of care		Effective communication	2 (Carroll‐Solomon & Denny, 2005; Francis‐Coad et al., 2018)
Treatment/care	76 (Al‐Oraibi et al., 2012; Andersson & Hjelm, 2017; Arain et al., 2016; Arfken et al., 2001; Baril et al., 2014; Barker et al., 2002, 2009; Blanchard et al., 2022; Capezuti et al., 2002, 2007; Carroll‐Solomon & Denny, 2005; Chen et al., 2022; Crespin et al., 2010; Desai, Williams, Greene, Pierson, Caprio, & Hansen, 2013a, 2013b; Desai, Williams, Greene, Pierson, & Hansen, 2013; DeSure et al., 2013; Dugré et al., 2021; Flynn et al., 2002; Francis‐Coad et al., 2018; Fuller et al., 2022; Gibson et al., 2008; Gil & Capelas, 2022; Gray‐Miceli et al., 2010; Greene et al., 2005, 2010, 2011; Gurwitz et al., 2005; Handler et al., 2004; Hansen et al., 2006, 2010; Hewitt et al., 2018; Johnson & Madan, 2017; Joyce, 2020; Kapoor et al., 2019; Kepner et al., 2022; Kim et al., 2017; Kobayashi & Sugai, 2006; Kosse et al., 2015; Lachs et al., 2016; Lane et al., 2014; Lee & Cho, 2022; Lord et al., 2003; Lundström et al., 2007; Mak et al., 2022; McCloskey et al., 2017; McDerby et al., 2019; McGrane et al., 2022; Milligan, 2012; Milligan et al., 2011; Mirolsky‐Scala & Kraemer, 2009; Neyens et al., 2009; O'Regan et al., 2022; Pierson et al., 2007; Rahim‐Jamal et al., 2008; Rask et al., 2007; Ray et al., 2002; Robinovitch et al., 2013; Shmueli et al., 2014; Sluggett, Chen, et al., 2020; Sluggett, Hopkins, et al., 2020; Smith et al., 2022; St Clair et al., 2021; Theodos, 2004; Tommasini et al., 2008; Toots et al., 2019; Verrue et al., 2011; Vlaeyen et al., 2021; Wabe, Seaman, et al., 2022; Wabe, Siette, et al., 2022; Wagner et al., 2005, 2008; White, 2001; Whitney et al., 2017; Wilson et al., 2011; Yang et al., 2015)	Good teamwork	1 (Gil & Capelas, 2022)
Care on admission	1 (Lane, 2009)	Directed to organization	
Transfer of care	1 (Desai et al., 2011)	Effective protocol available	8 (Francis‐Coad et al., 2018; Gray‐Miceli et al., 2010; Kobayashi & Sugai, 2006; Mak et al., 2022; McGrane et al., 2022; Theodos, 2004; Wabe, Siette, et al., 2022; Wagner et al., 2008)
Date of incident (reported)	17 (Capezuti et al., 2002, 2007; Crespin et al., 2010; Francis‐Coad et al., 2018; Greene et al., 2005, 2010, 2011; Johnson & Madan, 2017; Joyce, 2020; Kobayashi & Sugai, 2006; Kosse et al., 2015; Lachs et al., 2016; McCloskey et al., 2017; Theodos, 2004; Tommasini et al., 2008; Wabe, Seaman, et al., 2022; Wagner et al., 2005)	Product/equipment/device management & availability/accessibility	4 (Al‐Oraibi et al., 2012; Capezuti et al., 2002, 2007; Carroll‐Solomon & Denny, 2005)
Other care settings involved		Ameliorating actions	Number of studies
Other nursing facility/care home	4 (Flynn et al., 2002; Francis‐Coad et al., 2018; Gray‐Miceli et al., 2010; Tommasini et al., 2008)	Patient related	
General hospital	2 (Flynn et al., 2002; Tommasini et al., 2008)	Management of injury	3 (Francis‐Coad et al., 2018; Johnson & Madan, 2017; Joyce, 2020)
Community care facility	1 (Gray‐Miceli et al., 2010)	Organization related	
Disability service	1 (Francis‐Coad et al., 2018)	Education/training	2 (Francis‐Coad et al., 2018; Gray‐Miceli et al., 2010)

Contributing factors	Number of studies	Actions to reduce risk	Number of studies
Resources/organization management		Patient factors	
Protocols/policy/procedure/guideline availability/adequacy	12 (Al‐Oraibi et al., 2012; Carroll‐Solomon & Denny, 2005; Crespin et al., 2010; Desai, Williams, Greene, Pierson, & Hansen, 2013; Greene et al., 2005; Handler et al., 2004; Hansen et al., 2010; Lane, 2009; Lee & Cho, 2022; McGrane et al., 2022; Wagner, Castle, & Handler, 2013a; Whitney et al., 2017)	Provision of protocols/decision support	8 (Dugré et al., 2021; Francis‐Coad et al., 2018; Gray‐Miceli et al., 2010; Kobayashi & Sugai, 2006; Neyens et al., 2009; Sluggett, Chen, et al., 2020; Sluggett, Hopkins, et al., 2020; Wabe, Siette, et al., 2022)
Workload	12 (Blanchard et al., 2022; Carroll‐Solomon & Denny, 2005; Crespin et al., 2010; Desai, Williams, Greene, Pierson, & Hansen, 2013; Gil & Capelas, 2022; Greene et al., 2005; Hansen et al., 2010; Lane, 2009; McGrane et al., 2022; Pierson et al., 2007; Theodos, 2004; Wagner, Castle, & Handler, 2013a)	Provision of adequate care/support	4 (Joyce, 2020; Lundström et al., 2007; Toots et al., 2019; Whitney et al., 2017)
Organization of teams/people	3 (Blanchard et al., 2022; Gil & Capelas, 2022; Lee & Cho, 2022)	Provision of patient education/training	3 (DeSure et al., 2013; Hewitt et al., 2018; McGrane et al., 2022)
Human resource availability	1 (McCloskey et al., 2017)	Provision of monitoring equipment	2 (Al‐Oraibi et al., 2012; Carroll‐Solomon & Denny, 2005)
Staff factors		Provision of medication dispensing kit	1 (Fuller et al., 2022)
Performance	14 (Andersson & Hjelm, 2017; Arain et al., 2016; Barker et al., 2002; Capezuti et al., 2007; Carroll‐Solomon & Denny, 2005; Desai, Williams, Greene, Pierson, & Hansen, 2013; Fuller et al., 2022; Gray‐Miceli et al., 2010; Hansen et al., 2010; Lane, 2009; McGrane et al., 2022; O'Regan et al., 2022; Pierson et al., 2007; Verrue et al., 2011)	Staff factors	
Communication	11 (Andersson & Hjelm, 2017; Crespin et al., 2010; Desai, Williams, Greene, Pierson, & Hansen, 2013; Fuller et al., 2022; Gil & Capelas, 2022; Greene et al., 2005, 2010; Hansen et al., 2010; Lane, 2009; Lane et al., 2014; Wagner, Castle, & Handler, 2013a)	Training	9 (Dugré et al., 2021; Francis‐Coad et al., 2018; Gray‐Miceli et al., 2010; Kobayashi & Sugai, 2006; McGrane et al., 2022; Sluggett, Chen, et al., 2020; Sluggett, Hopkins, et al., 2020; Toots et al., 2019; Whitney et al., 2017)
Behaviour	10 (Arain et al., 2016; Carroll‐Solomon & Denny, 2005; Crespin et al., 2010; Gil & Capelas, 2022; Lee & Cho, 2022; McGrane et al., 2022; O'Regan et al., 2022; Pierson et al., 2007; Theodos, 2004; Verrue et al., 2011)	Availability of checklists/protocols/policies	5 (Carroll‐Solomon & Denny, 2005; Kapoor et al., 2019; Kim et al., 2017; Theodos, 2004; Wabe, Siette, et al., 2022)
Social	10 (Capezuti et al., 2007; Carroll‐Solomon & Denny, 2005; Crespin et al., 2010; Desai, Williams, Greene, Pierson, & Hansen, 2013; Gil & Capelas, 2022; Greene et al., 2005; Handler et al., 2004; Hansen et al., 2010; Lane, 2009; Lane et al., 2014)	Supervision/assistance	4 (Lundström et al., 2007; Neyens et al., 2009; O'Regan et al., 2022; Wagner et al., 2005)
Patient factors		Organization/environmental factors	
Cognitive	30 (Arfken et al., 2001; Barker et al., 2009; Blanchard et al., 2022; Capezuti et al., 2002; Capezuti et al., 2007; Carroll‐Solomon & Denny, 2005; Fuller et al., 2022; Gil & Capelas, 2022; Gray‐Miceli et al., 2010; Greene et al., 2005; Hewitt et al., 2018; Joyce, 2020; Kim et al., 2017; Kobayashi & Sugai, 2006; Kosse et al., 2015; Lane, 2009; Lord et al., 2003; Lundström et al., 2007; Mak et al., 2022; McGrane et al., 2022; Mirolsky‐Scala & Kraemer, 2009; Neyens et al., 2009; Robinovitch et al., 2013; Sluggett, Chen, et al., 2020; Sluggett, Hopkins, et al., 2020; Tommasini et al., 2008; Wabe, Seaman, et al., 2022; Wabe, Siette, et al., 2022; Wagner et al., 2008; Wilson et al., 2011)	Improving safety culture	21 (Al‐Oraibi et al., 2012; Dugré et al., 2021; Gil & Capelas, 2022; Gray‐Miceli et al., 2010; Johnson & Madan, 2017; Joyce, 2020; Kapoor et al., 2019; Kim et al., 2017; Kobayashi & Sugai, 2006; Lee & Cho, 2022; McGrane et al., 2022; Rask et al., 2007; Sluggett, Chen, et al., 2020; Sluggett, Hopkins, et al., 2020; Theodos, 2004; Toots et al., 2019; Wabe, Siette, et al., 2022; Wagner et al., 2005, 2008; Wagner, Castle, & Handler, 2013a; Whitney et al., 2017)
Behaviour	15 (Blanchard et al., 2022; Capezuti et al., 2002; Gil & Capelas, 2022; Gray‐Miceli et al., 2010; Joyce, 2020; Kim et al., 2017; Kosse et al., 2015; Lachs et al., 2016; Lord et al., 2003; Lundström et al., 2007; McGrane et al., 2022; Neyens et al., 2009; Rask et al., 2007; St Clair et al., 2021; Toots et al., 2019)	Performing risk assessment/root cause analyses	12 (Carroll‐Solomon & Denny, 2005; Francis‐Coad et al., 2018; Gray‐Miceli et al., 2010; Johnson & Madan, 2017; Kim et al., 2017; Kobayashi & Sugai, 2006; Lachs et al., 2016; Rask et al., 2007; Theodos, 2004; Wabe, Siette, et al., 2022; Wagner et al., 2005, 2008)
Performance	3 (DeSure et al., 2013; Mak et al., 2022; McCloskey et al., 2017)	Improved leadership/guidance	2 (Carroll‐Solomon & Denny, 2005; Gil & Capelas, 2022)
Social	3 (Barker et al., 2009; Gil & Capelas, 2022; Lord et al., 2003)	Managing physical environment needs	2 (Capezuti et al., 2002, 2007)
Disease	2 (Gray‐Miceli et al., 2010; St Clair et al., 2021)	Arranging ready access to protocols/policies/decision support	1 (Neyens et al., 2009)
Communication	1 (Lundström et al., 2007)	Current code/specifications/regulations being met	1 (Francis‐Coad et al., 2018)
Other factors		Making arrangements for access to a service	1 (Dugré et al., 2021)
Work/environment	15 (Barker et al., 2009; Capezuti et al., 2007; Carroll‐Solomon & Denny, 2005; DeSure et al., 2013; Gray‐Miceli et al., 2010; Kobayashi & Sugai, 2006; Kosse et al., 2015; Lane, 2009; Lee & Cho, 2022; Lord et al., 2003; McGrane et al., 2022; O'Regan et al., 2022; Robinovitch et al., 2013; Wabe, Seaman, et al., 2022; Wagner et al., 2008)	Matching of staff to tasks/skills	1 (McGrane et al., 2022)
Organizational	11 (Carroll‐Solomon & Denny, 2005; Gil & Capelas, 2022; Gray‐Miceli et al., 2010; Greene et al., 2005; Hansen et al., 2010; Lane, 2009; Lee & Cho, 2022; Sluggett, Chen, et al., 2020; Toots et al., 2019; Verrue et al., 2011; Wagner, Castle, & Handler, 2013a)	Agent/equipment factors	
External	4 (Crespin et al., 2010; Desai, Williams, Greene, Pierson, & Hansen, 2013; Greene et al., 2005; Hansen et al., 2010)	Provision of equipment	8 (Al‐Oraibi et al., 2012; Capezuti et al., 2002, 2007; Dugré et al., 2021; McGrane et al., 2022; Sluggett, Chen, et al., 2020; Sluggett, Hopkins, et al., 2020; Theodos, 2004)

Detection	Number of studies	Outcomes	Number of studies
People involved		Degree of harm (reported)	44 (Al‐Oraibi et al., 2012; Andersson & Hjelm, 2017; Arain et al., 2016; Arfken et al., 2001; Baril et al., 2014; Capezuti et al., 2002; Carroll‐Solomon & Denny, 2005; Crespin et al., 2010; Desai et al., 2011; Desai, Williams, Greene, Pierson, Caprio, & Hansen, 2013a, 2013b; Desai, Williams, Greene, Pierson, & Hansen, 2013; Dugré et al., 2021; Francis‐Coad et al., 2018; Fuller et al., 2022; Gray‐Miceli et al., 2010; Greene et al., 2005, 2010, 2011; Gurwitz et al., 2005; Handler et al., 2004; Joyce, 2020; Kapoor et al., 2019; Kobayashi & Sugai, 2006; Kosse et al., 2015; Lane, 2009; Lane et al., 2014; Mak et al., 2022; McGrane et al., 2022; Milligan, 2012; Milligan et al., 2011; O'Regan et al., 2022; Pierson et al., 2007; Rahim‐Jamal et al., 2008; Rask et al., 2007; Shmueli et al., 2014; Sjogren et al., 2015; St Clair et al., 2021; Tommasini et al., 2008; Toots et al., 2019; Wabe, Seaman, et al., 2022; Wagner et al., 2005; Wagner, Castle, Reid, & Stone, 2013; White, 2001)
Healthcare professional	32 (Barker et al., 2002, 2009; Desai, Williams, Greene, Pierson, Caprio, & Hansen, 2013a, 2013b; Desai, Williams, Greene, Pierson, & Hansen, 2013; Dugré et al., 2021; Flynn et al., 2002; Fuller et al., 2022; Gibson et al., 2008; Gray‐Miceli et al., 2010; Gurwitz et al., 2005; Handler et al., 2004; Johnson & Madan, 2017; Joyce, 2020; Kosse et al., 2015; Lundström et al., 2007; McGrane et al., 2022; Neyens et al., 2009; Pierson et al., 2007; Rahim‐Jamal et al., 2008; Shmueli et al., 2014; Sjogren et al., 2015; Sluggett, Chen, et al., 2020; Sluggett, Hopkins, et al., 2020; Theodos, 2004; Toots et al., 2019; Verrue et al., 2011; Vlaeyen et al., 2021; Wagner et al., 2005, 2008; Whitney et al., 2017)	Type of harm	
Healthcare worker	13 (Carroll‐Solomon & Denny, 2005; Chen et al., 2022; Gil & Capelas, 2022; Kapoor et al., 2019; Kosse et al., 2015; Lachs et al., 2016; Lundström et al., 2007; Pierson et al., 2007; Smith et al., 2022; St Clair et al., 2021; Theodos, 2004; Wagner, Castle, & Handler, 2013a; Wagner, Castle, Reid, & Stone, 2013)	Injury	28 (Al‐Oraibi et al., 2012; Andersson & Hjelm, 2017; Arfken et al., 2001; Capezuti et al., 2002, 2007; Carroll‐Solomon & Denny, 2005; Dugré et al., 2021; Francis‐Coad et al., 2018; Fuller et al., 2022; Gray‐Miceli et al., 2010; Johnson & Madan, 2017; Joyce, 2020; Kobayashi & Sugai, 2006; Kosse et al., 2015; Lachs et al., 2016; Mak et al., 2022; McGrane et al., 2022; Mirolsky‐Scala & Kraemer, 2009; O'Regan et al., 2022; Rask et al., 2007; Sjogren et al., 2015; St Clair et al., 2021; Theodos, 2004; Tommasini et al., 2008; Toots et al., 2019; Wabe, Seaman, et al., 2022; Wagner et al., 2005; White, 2001)
Another patient	1 (Smith et al., 2022)	Other	19 (Arain et al., 2016; Baril et al., 2014; Crespin et al., 2010; Desai et al., 2011; Desai, Williams, Greene, Pierson, Caprio, & Hansen, 2013a, 2013b; Desai, Williams, Greene, Pierson, & Hansen, 2013; Greene et al., 2005, 2010, 2011; Gurwitz et al., 2005; Handler et al., 2004; Lane, 2009; Lane et al., 2014; Milligan, 2012; Milligan et al., 2011; Pierson et al., 2007; Rahim‐Jamal et al., 2008; Shmueli et al., 2014)
Carer/home aide/assistant	1 (Gil & Capelas, 2022)	Pathophysiology and injury	2 (Kapoor et al., 2019; Wagner, Castle, Reid, & Stone, 2013)
Process		Social/economic impact (reported)	3 (Al‐Oraibi et al., 2012; McCloskey et al., 2017; White, 2001)
Error recognition	33 (Andersson & Hjelm, 2017; Barker et al., 2002; Chen et al., 2022; Crespin et al., 2010; Desai et al., 2011; Desai, Williams, Greene, Pierson, Caprio, & Hansen, 2013a, 2013b; Desai, Williams, Greene, Pierson, & Hansen, 2013; Dugré et al., 2021; Flynn et al., 2002; Fuller et al., 2022; Gil & Capelas, 2022; Greene et al., 2005, 2010, 2011; Gurwitz et al., 2005; Handler et al., 2004; Hansen et al., 2006, 2010; Lane, 2009; Lane et al., 2014; McDerby et al., 2019; McGrane et al., 2022; Milligan, 2012; Milligan et al., 2011; Pierson et al., 2007; Rahim‐Jamal et al., 2008; Shmueli et al., 2014; Sjogren et al., 2015; Smith et al., 2022; St Clair et al., 2021; Verrue et al., 2011; Wagner, Castle, & Handler, 2013a)	Organizational outcomes (reported)	1 (Wagner, Castle, Reid, & Stone, 2013)
By change in patient's status	11 (Barker et al., 2009; DeSure et al., 2013; Hewitt et al., 2018; Lundström et al., 2007; Mirolsky‐Scala & Kraemer, 2009; Neyens et al., 2009; Sluggett, Chen, et al., 2020; Sluggett, Hopkins, et al., 2020; Theodos, 2004; Toots et al., 2019; Whitney et al., 2017)		
By a count/audit/review	8 (Francis‐Coad et al., 2018; Gibson et al., 2008; Johnson & Madan, 2017; Joyce, 2020; Kapoor et al., 2019; Kosse et al., 2015; Rask et al., 2007; Wagner et al., 2005)		
Proactive risk assessment	5 (Capezuti et al., 2002, 2007; Gray‐Miceli et al., 2010; Kim et al., 2017; Kobayashi & Sugai, 2006)		
By machine/system/environmental change/alarm	2 (Al‐Oraibi et al., 2012; Carroll‐Solomon & Denny, 2005)		

#### 3.3.1 Incident types

The incident types most often reported included *medication/intravenous fluids* (Andersson & Hjelm, 2017; Arain et al., 2016; Baril et al., 2014; Barker et al., 2002; Carroll‐Solomon & Denny, 2005; Crespin et al., 2010; Desai et al., 2011; Desai, Williams, Greene, Pierson, Caprio, & Hansen, 2013a, 2013b; Desai, Williams, Greene, Pierson, & Hansen, 2013; Dugré et al., 2021; Flynn et al., 2002; Fuller et al., 2022; Gray‐Miceli et al., 2010; Greene et al., 2005, 2010, 2011; Gurwitz et al., 2005; Handler et al., 2004; Hansen et al., 2006, 2010; Kapoor et al., 2019; Lane, 2009; Lane et al., 2014; Lee & Cho, 2022; McDerby et al., 2019; Milligan, 2012; Milligan et al., 2011; Pierson et al., 2007; Rahim‐Jamal et al., 2008; Ray et al., 2002; Shmueli et al., 2014; Sluggett, Chen, et al., 2020; Sluggett, Hopkins, et al., 2020; St Clair et al., 2021; Verrue et al., 2011), *clinical process/procedure* (Al‐Oraibi et al., 2012; Andersson & Hjelm, 2017; Arain et al., 2016; Baril et al., 2014; Barker et al., 2002; Blanchard et al., 2022; Capezuti et al., 2002, 2007; Carroll‐Solomon & Denny, 2005; Crespin et al., 2010; Desai et al., 2011; Desai, Williams, Greene, Pierson, Caprio, & Hansen, 2013a, 2013b; Desai, Williams, Greene, Pierson, & Hansen, 2013; Dugré et al., 2021; Flynn et al., 2002; Fuller et al., 2022; Gil & Capelas, 2022; Greene et al., 2005, 2010, 2011; Gurwitz et al., 2005; Handler et al., 2004; Hansen et al., 2006, 2010; Kapoor et al., 2019; Lane, 2009; Lane et al., 2014; McDerby et al., 2019; McGrane et al., 2022; Milligan, 2012; Milligan et al., 2011; Pierson et al., 2007; Rahim‐Jamal et al., 2008; Shmueli et al., 2014; Sjogren et al., 2015; Sluggett, Hopkins, et al., 2020; White, 2001), and *patient accidents (falls)* (Al‐Oraibi et al., 2012; Andersson & Hjelm, 2017; Arfken et al., 2001; Barker et al., 2009; Capezuti et al., 2002, 2007; Carroll‐Solomon & Denny, 2005; DeSure et al., 2013; Francis‐Coad et al., 2018; Gibson et al., 2008; Gray‐Miceli et al., 2010; Hewitt et al., 2018; Johnson & Madan, 2017; Joyce, 2020; Kapoor et al., 2019; Kobayashi & Sugai, 2006; Kosse et al., 2015; Lee & Cho, 2022; Lord et al., 2003; Mak et al., 2022; Mirolsky‐Scala & Kraemer, 2009; Neyens et al., 2009; Rask et al., 2007; Ray et al., 2002; Robinovitch et al., 2013; Shmueli et al., 2014; Sjogren et al., 2015; St Clair et al., 2021; Theodos, 2004; Tommasini et al., 2008; Toots et al., 2019; Vlaeyen et al., 2021; Wabe, Seaman, et al., 2022; Wabe, Siette, et al., 2022; Wagner et al., 2005, 2008; Wagner, Castle, Reid, & Stone, 2013; Whitney et al., 2017; Wilson et al., 2011; Yang et al., 2015). Whilst falls were categorized as a patient accident within the framework, no other types of patient accidents were reported. Other incident types without data were *blood/blood products*, *nutrition*, and *oxygen/gas/vapour*. Amongst the medication/intravenous fluids studies, medication list was the most involved attribute (*n* = 27) (Arain et al., 2016; Baril et al., 2014; Barker et al., 2002; Carroll‐Solomon & Denny, 2005; Crespin et al., 2010; Desai et al., 2011; Desai, Williams, Greene, Pierson, Caprio, & Hansen, 2013a, 2013b; Desai, Williams, Greene, Pierson, & Hansen, 2013; Dugré et al., 2021; Fuller et al., 2022; Gray‐Miceli et al., 2010; Greene et al., 2010; Gurwitz et al., 2005; Handler et al., 2004; Hansen et al., 2006, 2010; Lee & Cho, 2022; McDerby et al., 2019; Milligan, 2012; Milligan et al., 2011; Pierson et al., 2007; Rahim‐Jamal et al., 2008; Ray et al., 2002; Shmueli et al., 2014; Sluggett, Chen, et al., 2020; Sluggett, Hopkins, et al., 2020), whilst there were no studies that included data on intravenous fluid lists. Medication processes most often reported in the literature included *administration* (*n* = 23) (Andersson & Hjelm, 2017; Arain et al., 2016; Baril et al., 2014; Barker et al., 2002; Crespin et al., 2010; Desai et al., 2011; Desai, Williams, Greene, Pierson, Caprio, & Hansen, 2013a, 2013b; Desai, Williams, Greene, Pierson, & Hansen, 2013; Dugré et al., 2021; Greene et al., 2005, 2010; Gurwitz et al., 2005; Handler et al., 2004; Lane, 2009; Lane et al., 2014; McDerby et al., 2019; Milligan, 2012; Milligan et al., 2011; Pierson et al., 2007; Shmueli et al., 2014), *prescribing* (*n* = 15) (Crespin et al., 2010; Desai et al., 2011; Desai, Williams, Greene, Pierson, Caprio, & Hansen, 2013a, 2013b; Desai, Williams, Greene, Pierson, & Hansen, 2013; Dugré et al., 2021; Greene et al., 2005, 2010; Hansen et al., 2006; Lane et al., 2014; Milligan, 2012; Pierson et al., 2007; Ray et al., 2002; Verrue et al., 2011), *preparation/dispensing* (*n* = 15) (Baril et al., 2014; Crespin et al., 2010; Desai, Williams, Greene, Pierson, Caprio, & Hansen, 2013a, 2013b; Desai, Williams, Greene, Pierson, & Hansen, 2013; Fuller et al., 2022; Greene et al., 2010; Gurwitz et al., 2005; Handler et al., 2004; Hansen et al., 2010; Lane et al., 2014; McDerby et al., 2019; Milligan, 2012; Milligan et al., 2011) and *monitoring* (*n* = 14) (Crespin et al., 2010; Desai et al., 2011; Desai, Williams, Greene, Pierson, Caprio, & Hansen, 2013a, 2013b; Desai, Williams, Greene, Pierson, & Hansen, 2013; Greene et al., 2005, 2010, 2011; Gurwitz et al., 2005; Hansen et al., 2006; Lane, 2009; Lane et al., 2014; Milligan, 2012; Pierson et al., 2007). Eleven of 12 potential medication problems in the WHO conceptual framework were identified, with the most common being *wrong dose/strength of frequency* (*n* = 23) (Andersson & Hjelm, 2017; Arain et al., 2016; Baril et al., 2014; Barker et al., 2002; Crespin et al., 2010; Desai et al., 2011; Desai, Williams, Greene, Pierson, Caprio, & Hansen, 2013a, 2013b; Desai, Williams, Greene, Pierson, & Hansen, 2013; Dugré et al., 2021; Flynn et al., 2002; Fuller et al., 2022; Greene et al., 2005, 2010, 2011; Gurwitz et al., 2005; Handler et al., 2004; Lane, 2009; Lane et al., 2014; McDerby et al., 2019; Milligan, 2012; Milligan et al., 2011; Pierson et al., 2007), *wrong drug* (*n* = 19) (Arain et al., 2016; Barker et al., 2002; Crespin et al., 2010; Desai et al., 2011; Desai, Williams, Greene, Pierson, Caprio, & Hansen, 2013a, 2013b; Desai, Williams, Greene, Pierson, & Hansen, 2013; Dugré et al., 2021; Greene et al., 2005, 2010, 2011; Gurwitz et al., 2005; Handler et al., 2004; Hansen et al., 2010; Lane, 2009; McDerby et al., 2019; Milligan, 2012; Milligan et al., 2011; Pierson et al., 2007), and *omitted medicine or dose* (*n* = 17) (Arain et al., 2016; Baril et al., 2014; Barker et al., 2002; Dugré et al., 2021; Flynn et al., 2002; Greene et al., 2005, 2010, 2011; Handler et al., 2004; Hansen et al., 2010; Lane, 2009; Lane et al., 2014; McDerby et al., 2019; Milligan, 2012; Milligan et al., 2011; Pierson et al., 2007; St Clair et al., 2021). In the *clinical process/procedure* category, procedural processes (*n* = 28) including treatment and intervention were most commonly reported in the literature (Arain et al., 2016; Baril et al., 2014; Barker et al., 2002; Capezuti et al., 2002, 2007; Crespin et al., 2010; Desai et al., 2011; Desai, Williams, Greene, Pierson, Caprio, & Hansen, 2013a, 2013b; Desai, Williams, Greene, Pierson, & Hansen, 2013; Flynn et al., 2002; Greene et al., 2005, 2010, 2011; Gurwitz et al., 2005; Handler et al., 2004; Hansen et al., 2006, 2010; Lane, 2009; Lane et al., 2014; McDerby et al., 2019; McGrane et al., 2022; Milligan, 2012; Milligan et al., 2011; Pierson et al., 2007; Rahim‐Jamal et al., 2008; Shmueli et al., 2014; Sluggett, Hopkins, et al., 2020). Problems with clinical processes/procedures included incomplete or inadequate clinical processes or procedures (*n* = 29) (Andersson & Hjelm, 2017; Baril et al., 2014; Barker et al., 2002; Blanchard et al., 2022; Capezuti et al., 2002, 2007; Carroll‐Solomon & Denny, 2005; Crespin et al., 2010; Desai et al., 2011; Desai, Williams, Greene, Pierson, Caprio, & Hansen, 2013a, 2013b; Dugré et al., 2021; Flynn et al., 2002; Fuller et al., 2022; Gil & Capelas, 2022; Greene et al., 2005, 2010, 2011; Gurwitz et al., 2005; Handler et al., 2004; Hansen et al., 2006, 2010; Lane, 2009; Lane et al., 2014; McGrane et al., 2022; Milligan, 2012; Milligan et al., 2011; Sjogren et al., 2015; White, 2001), wrong patient (*n* = 18) (Arain et al., 2016; Baril et al., 2014; Barker et al., 2002; Crespin et al., 2010; Desai et al., 2011; Desai, Williams, Greene, Pierson, Caprio, & Hansen, 2013a, 2013b; Desai, Williams, Greene, Pierson, & Hansen, 2013; Dugré et al., 2021; Flynn et al., 2002; Fuller et al., 2022; Greene et al., 2005, 2010; Handler et al., 2004; Lane, 2009; Lane et al., 2014; Milligan, 2012; Pierson et al., 2007), or clinical processes or procedures not performed when indicated (*n* = 15) (Baril et al., 2014; Barker et al., 2002; Crespin et al., 2010; Desai, Williams, Greene, Pierson, & Hansen, 2013; Dugré et al., 2021; Flynn et al., 2002; Gil & Capelas, 2022; Greene et al., 2005, 2010, 2011; Kapoor et al., 2019; Lane, 2009; Lane et al., 2014; McGrane et al., 2022; Pierson et al., 2007). For patient accidents (falls), the fall was often due to a loss of balance (*n* = 9) (Capezuti et al., 2002, 2007; Joyce, 2020; Lord et al., 2003; Mirolsky‐Scala & Kraemer, 2009; Robinovitch et al., 2013; Tommasini et al., 2008; Wagner et al., 2005; Yang et al., 2015), slip (*n* = 5) (Mirolsky‐Scala & Kraemer, 2009; Robinovitch et al., 2013; Tommasini et al., 2008; Wagner et al., 2005; Yang et al., 2015) or a stumble/trip (*n* = 4) (Mirolsky‐Scala & Kraemer, 2009; Robinovitch et al., 2013; Wagner et al., 2005; Yang et al., 2015). Equipment associated with falls included bed (*n* = 9) (Capezuti et al., 2002, 2007; Carroll‐Solomon & Denny, 2005; Kobayashi & Sugai, 2006; Lord et al., 2003; Neyens et al., 2009; Shmueli et al., 2014; Theodos, 2004; Wagner et al., 2005) and chair (*n* = 5) (Carroll‐Solomon & Denny, 2005; Kobayashi & Sugai, 2006; Lord et al., 2003; Robinovitch et al., 2013; Theodos, 2004).

#### 3.3.2 Incident characteristics

The majority of people involved in the origin of the incident were *healthcare professionals* (*n* = 33) (Andersson & Hjelm, 2017; Arain et al., 2016; Baril et al., 2014; Barker et al., 2002; Desai et al., 2011; Desai, Williams, Greene, Pierson, Caprio, & Hansen, 2013a; Desai, Williams, Greene, Pierson, & Hansen, 2013; Dugré et al., 2021; Flynn et al., 2002; Fuller et al., 2022; Gray‐Miceli et al., 2010; Greene et al., 2005, 2010, 2011; Gurwitz et al., 2005; Handler et al., 2004; Hansen et al., 2006, 2010; Johnson & Madan, 2017; Kobayashi & Sugai, 2006; Lane, 2009; Lane et al., 2014; McDerby et al., 2019; Mirolsky‐Scala & Kraemer, 2009; Pierson et al., 2007; Rahim‐Jamal et al., 2008; Sluggett, Chen, et al., 2020; Sluggett, Hopkins, et al., 2020; Toots et al., 2019; Verrue et al., 2011; Wagner et al., 2008; Wagner, Castle, & Handler, 2013a; White, 2001) or *healthcare workers* (*n* = 10) (Blanchard et al., 2022; Gil & Capelas, 2022; Kosse et al., 2015; Lee & Cho, 2022; Lord et al., 2003; Lundström et al., 2007; McCloskey et al., 2017; McGrane et al., 2022; Smith et al., 2022; Wilson et al., 2011), with only three studies reporting *another patient* being involved (Joyce, 2020; Lundström et al., 2007; Robinovitch et al., 2013). Of the healthcare professionals, nurses, pharmacists and healthcare assistants were most often reported to be involved in incidents. The most reported stage of care where the incident occurred was during *treatment* (*n* = 76) (Al‐Oraibi et al., 2012; Andersson & Hjelm, 2017; Arain et al., 2016; Arfken et al., 2001; Baril et al., 2014; Barker et al., 2002, 2009; Blanchard et al., 2022; Capezuti et al., 2002, 2007; Carroll‐Solomon & Denny, 2005; Chen et al., 2022; Crespin et al., 2010; Desai, Williams, Greene, Pierson, Caprio, & Hansen, 2013a, 2013b; Desai, Williams, Greene, Pierson, & Hansen, 2013; DeSure et al., 2013; Dugré et al., 2021; Flynn et al., 2002; Francis‐Coad et al., 2018; Fuller et al., 2022; Gibson et al., 2008; Gil & Capelas, 2022; Gray‐Miceli et al., 2010; Greene et al., 2005, 2010, 2011; Gurwitz et al., 2005; Handler et al., 2004; Hansen et al., 2006, 2010; Hewitt et al., 2018; Johnson & Madan, 2017; Joyce, 2020; Kapoor et al., 2019; Kepner et al., 2022; Kim et al., 2017; Kobayashi & Sugai, 2006; Kosse et al., 2015; Lachs et al., 2016; Lane et al., 2014; Lee & Cho, 2022; Lord et al., 2003; Lundström et al., 2007; Mak et al., 2022; McCloskey et al., 2017; McDerby et al., 2019; McGrane et al., 2022; Milligan, 2012; Milligan et al., 2011; Mirolsky‐Scala & Kraemer, 2009; Neyens et al., 2009; O'Regan et al., 2022; Pierson et al., 2007; Rahim‐Jamal et al., 2008; Rask et al., 2007; Ray et al., 2002; Robinovitch et al., 2013; Shmueli et al., 2014; Sluggett, Chen, et al., 2020; Sluggett, Hopkins, et al., 2020; Smith et al., 2022; St Clair et al., 2021; Theodos, 2004; Tommasini et al., 2008; Toots et al., 2019; Verrue et al., 2011; Vlaeyen et al., 2021; Wabe, Seaman, et al., 2022; Wabe, Siette, et al., 2022; Wagner et al., 2005, 2008; White, 2001; Whitney et al., 2017; Wilson et al., 2011; Yang et al., 2015), whilst the *date of incident* was reported in 17 studies (Capezuti et al., 2002, 2007; Crespin et al., 2010; Francis‐Coad et al., 2018; Greene et al., 2005, 2010, 2011; Johnson & Madan, 2017; Joyce, 2020; Kobayashi & Sugai, 2006; Kosse et al., 2015; Lachs et al., 2016; McCloskey et al., 2017; Theodos, 2004; Tommasini et al., 2008; Wabe, Seaman, et al., 2022; Wagner et al., 2005). Only four studies directly mentioned *other care settings* being involved in the incidents, all of which included *other nursing facilities or care homes* (Flynn et al., 2002; Francis‐Coad et al., 2018; Gray‐Miceli et al., 2010; Tommasini et al., 2008), two included general hospitals (Flynn et al., 2002; Tommasini et al., 2008), one each included a community care facility (Gray‐Miceli et al., 2010) or disability service (Francis‐Coad et al., 2018).

#### 3.3.3 Detection of incidents

*Error recognitio*n (*n* = 33) (Andersson & Hjelm, 2017; Barker et al., 2002; Chen et al., 2022; Crespin et al., 2010; Desai et al., 2011; Desai, Williams, Greene, Pierson, Caprio, & Hansen, 2013a, 2013b; Desai, Williams, Greene, Pierson, & Hansen, 2013; Dugré et al., 2021; Flynn et al., 2002; Fuller et al., 2022; Gil & Capelas, 2022; Greene et al., 2005, 2010, 2011; Gurwitz et al., 2005; Handler et al., 2004; Hansen et al., 2006, 2010; Lane, 2009; Lane et al., 2014; McDerby et al., 2019; McGrane et al., 2022; Milligan, 2012; Milligan et al., 2011; Pierson et al., 2007; Rahim‐Jamal et al., 2008; Shmueli et al., 2014; Sjogren et al., 2015; Smith et al., 2022; St Clair et al., 2021; Verrue et al., 2011; Wagner, Castle, & Handler, 2013a), where staff recognize an error has occurred, was the highest frequency process for detection of incidents, followed by a *change in patient's status* (*n* = 11) (Barker et al., 2009; DeSure et al., 2013; Hewitt et al., 2018; Lundström et al., 2007; Mirolsky‐Scala & Kraemer, 2009; Neyens et al., 2009; Sluggett, Chen, et al., 2020; Sluggett, Hopkins, et al., 2020; Theodos, 2004; Toots et al., 2019; Whitney et al., 2017), some form of *counting*, *audit or review* (*n* = 8) (Francis‐Coad et al., 2018; Gibson et al., 2008; Johnson & Madan, 2017; Joyce, 2020; Kapoor et al., 2019; Kosse et al., 2015; Rask et al., 2007; Wagner et al., 2005) and *proactive risk assessment* (*n* = 5) (Capezuti et al., 2002, 2007; Gray‐Miceli et al., 2010; Kim et al., 2017; Kobayashi & Sugai, 2006). Two studies reported a process of using a *machine*, *system*, *environmental change or alarm* for the detection of incidents (Al‐Oraibi et al., 2012; Carroll‐Solomon & Denny, 2005).

#### 3.3.4 Contributing factors

Contributing factors were identified across four categories; *resource and organization management*, *staff factors*, *patients factors* and *other factors*. Protocols, policy, procedure, guidelines availability/adequacy (*n* = 12) (Al‐Oraibi et al., 2012; Carroll‐Solomon & Denny, 2005; Crespin et al., 2010; Desai, Williams, Greene, Pierson, & Hansen, 2013; Greene et al., 2005; Handler et al., 2004; Hansen et al., 2010; Lane, 2009; Lee & Cho, 2022; McGrane et al., 2022; Wagner, Castle, & Handler, 2013a; Whitney et al., 2017) and workload (*n* = 12) (Blanchard et al., 2022; Carroll‐Solomon & Denny, 2005; Crespin et al., 2010; Desai, Williams, Greene, Pierson, & Hansen, 2013; Gil & Capelas, 2022; Greene et al., 2005; Hansen et al., 2010; Lane, 2009; McGrane et al., 2022; Pierson et al., 2007; Theodos, 2004; Wagner, Castle, & Handler, 2013a) were the most commonly identified contributing factors within resource and organization management. Performance (*n* = 14) (Andersson & Hjelm, 2017; Arain et al., 2016; Barker et al., 2002; Capezuti et al., 2007; Carroll‐Solomon & Denny, 2005; Desai, Williams, Greene, Pierson, & Hansen, 2013; Fuller et al., 2022; Gray‐Miceli et al., 2010; Hansen et al., 2010; Lane, 2009; McGrane et al., 2022; O'Regan et al., 2022; Pierson et al., 2007; Verrue et al., 2011) and communication (*n* = 11) (Andersson & Hjelm, 2017; Crespin et al., 2010; Desai, Williams, Greene, Pierson, & Hansen, 2013; Fuller et al., 2022; Gil & Capelas, 2022; Greene et al., 2005, 2010; Hansen et al., 2010; Lane, 2009; Lane et al., 2014; Wagner, Castle, & Handler, 2013a) were prominent staff contributory factors, whereas amongst patient contributory factors it was cognitive (*n* = 30) (Arfken et al., 2001; Barker et al., 2009; Blanchard et al., 2022; Capezuti et al., 2002, 2007; Carroll‐Solomon & Denny, 2005; Fuller et al., 2022; Gil & Capelas, 2022; Gray‐Miceli et al., 2010; Greene et al., 2005; Hewitt et al., 2018; Joyce, 2020; Kim et al., 2017; Kobayashi & Sugai, 2006; Kosse et al., 2015; Lane, 2009; Lord et al., 2003; Lundström et al., 2007; Mak et al., 2022; McGrane et al., 2022; Mirolsky‐Scala & Kraemer, 2009; Neyens et al., 2009; Robinovitch et al., 2013; Sluggett, Chen, et al., 2020; Sluggett, Hopkins, et al., 2020; Tommasini et al., 2008; Wabe, Seaman, et al., 2022; Wabe, Siette, et al., 2022; Wagner et al., 2008; Wilson et al., 2011) and behavioural factors (*n* = 15) (Blanchard et al., 2022; Capezuti et al., 2002; Gil & Capelas, 2022; Gray‐Miceli et al., 2010; Joyce, 2020; Kim et al., 2017; Kosse et al., 2015; Lachs et al., 2016; Lord et al., 2003; Lundström et al., 2007; McGrane et al., 2022; Neyens et al., 2009; Rask et al., 2007; St Clair et al., 2021; Toots et al., 2019) that were most prominent. Other contributory factors were the work/environment (*n* = 13) (Barker et al., 2009; Capezuti et al., 2007; Carroll‐Solomon & Denny, 2005; DeSure et al., 2013; Gray‐Miceli et al., 2010; Kobayashi & Sugai, 2006; Kosse et al., 2015; Lane, 2009; Lee & Cho, 2022; Lord et al., 2003; McGrane et al., 2022; O'Regan et al., 2022; Robinovitch et al., 2013; Wabe, Seaman, et al., 2022; Wagner et al., 2008) and organizational factors (*n* = 11) (Carroll‐Solomon & Denny, 2005; Gil & Capelas, 2022; Gray‐Miceli et al., 2010; Greene et al., 2005; Hansen et al., 2010; Lane, 2009; Lee & Cho, 2022; Sluggett, Chen, et al., 2020; Toots et al., 2019; Verrue et al., 2011; Wagner, Castle, & Handler, 2013a), with only four studies identifying external factors contributing to incidents (Crespin et al., 2010; Desai, Williams, Greene, Pierson, & Hansen, 2013; Greene et al., 2005; Hansen et al., 2010).

#### 3.3.5 Mitigating factors

Only a small number of studies reported on the factors that helped to mitigate harm once an incident had occurred. These included having some form of management, treatment or care undertaken (*n* = 9) (Al‐Oraibi et al., 2012; DeSure et al., 2013; Francis‐Coad et al., 2018; Gray‐Miceli et al., 2010; Joyce, 2020; Lundström et al., 2007; Mak et al., 2022; Neyens et al., 2009; Sluggett, Hopkins, et al., 2020), an effective protocol available (*n* = 9) (Francis‐Coad et al., 2018; Gray‐Miceli et al., 2010; Kobayashi & Sugai, 2006; Mak et al., 2022; McGrane et al., 2022; Theodos, 2004; Wabe, Siette, et al., 2022; Wagner et al., 2008), and having a product, equipment or device either available or accessible (*n* = 4) (Al‐Oraibi et al., 2012; Capezuti et al., 2002, 2007; Carroll‐Solomon & Denny, 2005).

#### 3.3.6 Ameliorating actions

Very few studies reported data on ameliorating actions; three studies included patient‐related actions that consisted of managing the injury (Francis‐Coad et al., 2018; Johnson & Madan, 2017; Joyce, 2020), and two included organization‐related actions of education or training (Francis‐Coad et al., 2018; Gray‐Miceli et al., 2010).

#### 3.3.7 Actions taken to reduce risk

Actions taken to reduce risk of incidents included staff factors, organization or environment factors, agent or equipment factors and patient factors. Within these, *improving safety culture* (*n* = 21) (Al‐Oraibi et al., 2012; Dugré et al., 2021; Gil & Capelas, 2022; Gray‐Miceli et al., 2010; Johnson & Madan, 2017; Joyce, 2020; Kapoor et al., 2019; Kim et al., 2017; Kobayashi & Sugai, 2006; Lee & Cho, 2022; McGrane et al., 2022; Rask et al., 2007; Sluggett, Chen, et al., 2020; Sluggett, Hopkins, et al., 2020; Theodos, 2004; Toots et al., 2019; Wabe, Siette, et al., 2022; Wagner et al., 2005, 2008; Wagner, Castle, & Handler, 2013a; Whitney et al., 2017) *staff training* (*n* = 10) (Dugré et al., 2021; Francis‐Coad et al., 2018; Gray‐Miceli et al., 2010; Kobayashi & Sugai, 2006; McGrane et al., 2022; Sluggett, Chen, et al., 2020; Sluggett, Hopkins, et al., 2020; Toots et al., 2019; Whitney et al., 2017), performing risk assessments or root cause analyses (Carroll‐Solomon & Denny, 2005; Francis‐Coad et al., 2018; Gray‐Miceli et al., 2010; Johnson & Madan, 2017; Kim et al., 2017; Kobayashi & Sugai, 2006; Lachs et al., 2016; Rask et al., 2007; Theodos, 2004; Wabe, Siette, et al., 2022; Wagner et al., 2005, 2008), and *provision of equipment* (*n* = 8) (Al‐Oraibi et al., 2012; Capezuti et al., 2002, 2007; Dugré et al., 2021; McGrane et al., 2022; Sluggett, Chen, et al., 2020; Sluggett, Hopkins, et al., 2020; Theodos, 2004) were the highest reported actions taken to reduce risk. The most commonly reported patient factor was the *provision of protocols or decision support* (*n* = 8) (Dugré et al., 2021; Francis‐Coad et al., 2018; Gray‐Miceli et al., 2010; Kobayashi & Sugai, 2006; Neyens et al., 2009; Sluggett, Chen, et al., 2020; Sluggett, Hopkins, et al., 2020; Wabe, Siette, et al., 2022).

#### 3.3.8 Outcomes

Degree of harm (*n* = 44) (Al‐Oraibi et al., 2012; Andersson & Hjelm, 2017; Arain et al., 2016; Arfken et al., 2001; Baril et al., 2014; Capezuti et al., 2002; Carroll‐Solomon & Denny, 2005; Crespin et al., 2010; Desai et al., 2011; Desai, Williams, Greene, Pierson, Caprio, & Hansen, 2013a, 2013b; Desai, Williams, Greene, Pierson, & Hansen, 2013; Dugré et al., 2021; Francis‐Coad et al., 2018; Fuller et al., 2022; Gray‐Miceli et al., 2010; Greene et al., 2005, 2010, 2011; Gurwitz et al., 2005; Handler et al., 2004; Joyce, 2020; Kapoor et al., 2019; Kobayashi & Sugai, 2006; Kosse et al., 2015; Lane, 2009; Lane et al., 2014; Mak et al., 2022; McGrane et al., 2022; Milligan, 2012; Milligan et al., 2011; O'Regan et al., 2022; Pierson et al., 2007; Rahim‐Jamal et al., 2008; Rask et al., 2007; Shmueli et al., 2014; Sjogren et al., 2015; St Clair et al., 2021; Tommasini et al., 2008; Toots et al., 2019; Wabe, Seaman, et al., 2022; Wagner et al., 2005; Wagner, Castle, Reid, & Stone, 2013; White, 2001) was the most reported outcome following safety incidents. Where type of harm was reported, this most often consisted of injury to the patient (*n* = 28) (Al‐Oraibi et al., 2012; Andersson & Hjelm, 2017; Arfken et al., 2001; Capezuti et al., 2002, 2007; Carroll‐Solomon & Denny, 2005; Dugré et al., 2021; Francis‐Coad et al., 2018; Fuller et al., 2022; Gray‐Miceli et al., 2010; Johnson & Madan, 2017; Joyce, 2020; Kobayashi & Sugai, 2006; Kosse et al., 2015; Lachs et al., 2016; Mak et al., 2022; McGrane et al., 2022; Mirolsky‐Scala & Kraemer, 2009; O'Regan et al., 2022; Rask et al., 2007; Sjogren et al., 2015; St Clair et al., 2021; Theodos, 2004; Tommasini et al., 2008; Toots et al., 2019; Wabe, Seaman, et al., 2022; Wagner et al., 2005; White, 2001). Only three studies reported on any social or economic impact (Al‐Oraibi et al., 2012; McCloskey et al., 2017; White, 2001), and only one study reported on organizational outcomes (Wagner, Castle, Reid, & Stone, 2013).

## 4 DISCUSSION

This systematic review of 106 studies provides insight into the diverse systems and processes used to report safety incidents in care homes internationally, as well as the types of incidents studied. Despite the large number of studies included in this review, all were based on data from high‐income countries; another recent scoping review of care home regulation also only identified only studies based in high‐income countries (Pot et al., 2024) highlighting a knowledge gap around the quality of care in care homes in lower and middle‐income countries. This bias towards developed countries was present over a decade ago, and little appears to have changed (Lloyd‐Sherlock, 2014). We identified that nurses were responsible for incident reporting more than any other profession or role, reflecting the prominent role that nurses take in safety work where management priorities may be elsewhere (Andersson & Hjelm, 2017). It was unclear why nurses were most involved in incident reporting. Could be due to being more skilled in incident identification particularly where, for example, they play a more prominent role in creating documentation or in administering medications, or in broader adherence to safety principles (Vaismoradi, Tella, et al., 2020). Alternatively, there could be a hierarchal aspect to reporting, which could result in filtering reports (Macrae, 2016).

We found there is no standard reporting system used in care homes, though incident reporting systems and processes do share many common features, suggesting that there is an opportunity to develop more standardized systems to improve cross‐organizational learning. Papers highlighted the use of a formal, electronic computerized reporting system, as opposed to paper‐based reporting of incidents or hybrid systems, which matches incident reporting systems in primary and secondary care that can reduce the burden associated with managing paper systems, such as data entry (Al‐Rayes et al., 2020; Höcherl et al., 2022). Whilst electronic incident reporting systems were the most common amongst studies included in this review, it is likely that reporting bias contributed to this finding; paper‐based incident reporting systems can produce more inconsistent data and less accurate analyses (Sutejo et al., 2021), and therefore may be less likely to be studied. Incident reporting systems in care homes collected a wide range of information, which tended to differ depending on the incident type. Core information that was captured related to the resident, the incident to understand what caused the incident, the severity and injuries sustained, and the actions taken to prevent the incident from reoccurring, though again the data captured via incident reporting systems were heterogenous. Research is currently underway to develop a minimum dataset for care homes which includes components on the quality and safety of care (Burton et al., 2022), with care homes reportedly keen to share data for quality improvement despite concerns about data protection (Hanratty et al., 2023), and the findings from this review can be used to inform a minimum dataset for incident reporting in care homes, depending on incident types.

This review also provided insight into the types of incidents that are commonly reported in care homes using safety incident reporting systems. The most frequently reported incident categories were *patient behaviour*, *clinical process/procedure*, *documentation*, *medication/intravenous fluids*, and *falls*, which broadly reflects previous evidence (St Clair et al., 2022). The comparison with existing literature highlights how safety incident reporting, when data are synthesized, can be a useful tool for identifying the types of safety incidents that occur in care homes, though the previously reported challenges around using incident reporting as an epidemiological tool, largely due to biases in staff reporting, are acknowledged (Hamed & Konstantinidis, 2021; Howell et al., 2017; Macrae, 2016; Waring et al., 2016). It is also possible that other approaches can be used to identify incidents in care homes, such as through interviews with staff, residents and carers, and by using routinely collected data for further analysis such as medical record review or alternative approaches identified by studying high‐reliability organizations (Serou et al., 2021). These may provide a different lens on the types of incidents that occur in care homes, though this review was not designed to identify these. Notably, we identified only one study that examined safety reporting on admission (Lane, 2009), and one study that examined safety reporting in relation to transfer of care (Desai et al., 2011), suggesting either a lack of safety incident reporting in these contexts, or a lack of research. Regardless of which, this is an area that needs further examination, especially when both transfers and admissions are known to be high in risk (Kapoor et al., 2019), particularly during the COVID‐19 pandemic (Newman et al., 2023).

Various contributing and mitigating factors and actions to reduce risk were identified. The most reported action to reduce risk was to improve safety culture, which is a somewhat nebulous concept in healthcare settings but is additionally complex in care homes as they are people's homes (Rand et al., 2021) that requires more of a balance between risk and autonomy than in healthcare settings (Evans et al., 2018). Despite a number of tools being available to measure safety culture in care homes (Kim et al., 2022), safety culture in care homes is still relatively poorly understood (Gartshore et al., 2017), and the large variation in care home contexts and approaches to reporting safety incidents identified in this review suggests that a single, internationally‐applicable measure of safety culture for care homes would likely be difficult to achieve. We also identified that the detection of incidents was often via healthcare professionals or healthcare workers, with only one study obtaining data from another patient or resident (Smith et al., 2022). Residents' views of safety are important because of it being their own home (Rand et al., 2021), so further research is needed to examine how residents can contribute data on safety, including how such data could then be used to improve care quality. Individual outcomes following a safety incident were often reported, but the social and economic impact of incidents and organizational outcomes were rarely reported. The economic impact of patient safety in acute healthcare settings is considered to be significant, though challenges associated with capturing safety data make it difficult to produce accurate estimates. For instance one recent study of medication errors in acute settings in England is thought to cost around £100 m/year and contribute to 180,000 increased bed days (Rachel Ann et al., 2021). No studies are known to us that attempt to develop a broad understanding of the social and economic costs of all safety incidents in care homes.

Finally, the need to explore contributing factors as part of developing and implementing safety approaches has been highlighted as the top research priority in nursing homes (Simmons et al., 2016). In our review we were able to identify several patient, staff and organizational factors that contributed to reported safety incidents. Overwhelmingly, cognition was most reported patient contributing factor, likely reflecting the population group being studied and providing support for the development of safety interventions in care home settings that address cognitive factors. Safety interventions that target patient cognition, such as technology to monitor patients with dementia, need to be investigated in the context of potentially reducing patient autonomy (Hall et al., 2017). After patient cognition, the next highest contributing factor was patient behaviour, followed by organizational issues such as protocols, policies and guidelines and workload challenges. Staff contributing factors included performance and communication, reaffirming the importance of training to improve competencies and teamwork (Garay et al., 2023).

### 4.1 Strengths and limitations

This systematic review has several notable strengths. Firstly, the review team had a range of topic expertise including patient safety, health and wellbeing and methodological expertise in completing systematic reviews which enabled an extensive and comprehensive search to be completed. In addition, this is the first known review that has identified and synthesized data on incident reporting systems and processes in care homes that draw on international literature. This means a wealth of data has been analysed, and thus, the conclusions made encapsulate the diversity of the care home sector, as well as different contexts and countries. However, it is important to note some limitations. We only reported the processes and methods of reporting incidents that were described in the literature, with most papers providing little explanation of reporting practices. Therefore, underreporting may exist and although attempted, the results presented in this paper may not reflect the nuances of reporting practice that happen, specifically where incident reporting and learning practices occur outside of research studies. There was also a possibility for interpretation bias, specifically in relation to the terms and phrases used in the included papers. For example, some papers specifically documented who reported incidents including ‘nurses’ or ‘nursing staff’, yet little to no explanation of the difference or overlap was provided. Therefore, the research team had to interpret this information and apply judgement to the most suitable category to assign data to.

### 4.2 Recommendations for further research

Whilst we could make many recommendations for further research, we have opted to highlight three which we believe to be the most important. Firstly, there is a need to create a deeper understanding of how safety culture is developed and contributes to incident reporting in care homes, including the role of nurses and others in creating a culture of organizational learning. With this comes the need to better understand where responsibility for detection and reporting of safety incidents in care homes, in particular how care home staff can be supported to learn from and respond to safety incidents.

Secondly, outside of North American and Europe there is a pressing need for research that examines safety incident reporting in care homes, especially in low‐ and middle‐income countries but also in other developed countries where research is currently lacking. Such research will fill a gap in the evidence base by examining new contexts, including different funding sources, regulatory systems and types of care provided by care homes (Fischer et al., 2022).

Our final recommendation is that there is a need to examine how safety incident reporting, and research studies using safety incident reporting data, can better capture the wider social and economic costs of safety incidents. Doing so can help organizations to better understand the impact of safety incidents not just within the care home(s) but across the wider health and social care system. Care homes operate within complex health and social care systems, meaning that safety incidents are also likely to impact on healthcare services including emergency, primary and acute care. Development of this broader understanding of safety incidents that impact on other services, or even where other services are contributors to incidents that then span across multiple services, could help to inform cross‐system learning responses.

### 4.3 Implications for policy and practice

Nurses have a significant role to play in care home safety incident reporting, in keeping with their overall focus on safety work and safety leadership within care home settings (Johannessen et al., 2020; Prang & Jelsness‐Jorgensen, 2014) In some countries and settings, this will likely be reinforced by statutory and regulatory obligations, however not all care homes, particularly residential homes, will have access to regular nursing care. Where this is the case, the responsibility for incident reporting may fall onto others who should receive adequate training and support for reporting, managing and learning from safety incidents.

Our review findings highlight a need to ensure standard reporting practice across the care home sector, including the types of data captured in safety incident reports. Whilst incident reporting data is somewhat unreliable for epidemiological purposes (Macrae, 2016), efforts should still be made to improve its reliability to better inform practice, policy and research within the care home sector. Additionally, there should be standard practice for using incident reports to improve and inform resident care, including learning from incidents, which includes providing feedback to the staff involved, but also the resident and family of the person.

## 5 CONCLUSION

This systematic review has identified a complex picture of incident reporting in care homes, with the evidence base notably limited to high‐income countries, highlighting a significant knowledge gap. Several types of incidents contribute heavily to the literature, including falls, medication issues, documentation, patient behaviour and more general clinical processes and procedures. The findings emphasize the central role of nursing staff in reporting safety incidents and the lack of standardized reporting systems and processes. Future research should explore how safety cultures contribute to safety incident reporting in care homes and develop an understanding of how to better capture the wider social and economic impacts of safety incidents including how safety incident reporting can be used to both capture and demonstrate the costs for the wider health and social care system.

## AUTHOR CONTRIBUTIONS

The study was conceived by JS, JW, MS, LY‐M and PD and was overseen by JS. KS performed the searches and all primary screening. KB, PD, JW, LY‐M, CM and CN were involved in double screening. KS, JS and CM completed data extraction. CM and CN completed the quality assessment. All authors contributed to, read, provided feedback on and approved the final manuscript.

## FUNDING INFORMATION

This work is funded by The Dunhill Medical Trust, grant number RPGF2006\226.

## CONFLICT OF INTEREST STATEMENT

The authors declare no conflicts of interest.

### PEER REVIEW

The peer review history for this article is available at https://www.webofscience.com/api/gateway/wos/peer‐review/10.1111/jan.16264.

## Supporting information


Data S1.

Data S2.

## Data Availability

The data that support the findings of this study are available from the corresponding author upon reasonable request.
