# Supplementary material for: Systematic review of types of safety incidents and the processes and systems used for safety incident reporting in care homes
Source: J Adv Nurs. 2024 Jun 19;81(1):69–115. doi: 10.1111/jan.16264 (PMC11638520; doi:10.1111/jan.16264)
Supplement: Supplementary file 1 — Data S1. [file JAN-81-69-s002.docx]

| **EBSCO - CINAHL, MEDLINE and PsycINFO** | |
| --- | --- |
| S1 | "incident report*" OR "error report*" OR "incidents report*" OR "errors report*" |
| S2 | "care home*" OR "nursing home*" OR "long term care*" OR “residential*” |
| S3 | S1 AND S2 |
| S4 | ("Medical Error*" OR "Diagnostic Error*" OR "Medication Error*" OR "drug error*" OR "Observer Variation" OR "Patient safety" OR incident OR incidents OR "adverse event*" OR "near miss" OR "Risk Management" OR "Safety Management" OR Accident OR Accidents OR "Accident Prevention" OR Safety OR complication) AND ("Data Collection" OR "medical record*" OR "Health Record*" OR "report* system*" OR "anonymous report*" OR "non-anonymous report*" OR "voluntary report*" OR reporting) |
| S5 | S2 AND S4 |
| S6 | S3 OR S5 |
| **OVID - EMBASE 1996-2021, HMIC** | |
| S1 | ("incident report*" OR "error report*" OR "incidents report*" OR "errors report*").mp. [mp=ti, ab, hw, tn, ot, dm, mf, dv, kw, fx, dg] |
| S2 | ("care home*" OR "nursing home*" OR "long term care*").mp. [mp=ti, ab, hw, tn, ot, dm, mf, dv, kw, fx, dg] |
| S3 | Residential*.mp. [mp=ti, ab, hw, tn, ot, dm, mf, dv, kw, fx, dg] |
| S4 | S2 OR S3 |
| S5 | S1 AND S4 |
| S6 | ("Medical Error*" OR "Diagnostic Error*" OR "Medication Error*" OR "drug error*" OR "Observer Variation" OR "Patient safety" OR incident OR incidents OR "adverse event*" OR "near miss" OR "Risk Management" OR "Safety Management" OR Accident OR Accidents OR "Accident Prevention" OR Safety OR complication) .mp. [mp=ti, ab, hw, tn, ot, dm, mf, dv, kw, fx, dg] |
| S7 | ("Data Collection" OR "medical record*" OR "Health Record*" OR "report* system*" OR "anonymous report*" OR "non-anonymous report*" OR "voluntary report*" OR reporting).mp. [mp=ti, ab, hw, tn, ot, dm, mf, dv, kw, fx, dg] |
| S8 | S6 AND S7 |
| S9 | S5 AND S8 |
| S10 | S5 OR S9 |
| S11 | Limit S11 to english language |
| **Proquest – ASSISA** | |
| S1 | noft("incident report*" OR "error report*" OR "incidents report*" OR "errors report*") |
| S2 | noft("care home*" OR "nursing home*" OR "long term care*" OR “residential*”) |
| S3 | noft("incident report*" OR "error report*" OR "incidents report*" OR "errors report*") AND noft("care home*" OR "nursing home*" OR "long term care*" OR “residential*”) |
| S4 | ("Medical Error*" OR "Diagnostic Error*" OR "Medication Error*" OR "drug error*" OR "Observer Variation" OR "Patient safety" OR incident OR incidents OR "adverse event*" OR "near miss" OR "Risk Management" OR "Safety Management" OR Accident OR Accidents OR "Accident Prevention" OR Safety OR complication) AND ("Data Collection" OR "medical record*" OR "Health Record*" OR "report* system*" OR "anonymous report*" OR "non-anonymous report*" OR "voluntary report*" OR reporting) |
| S5 | noft("care home*" OR "nursing home*" OR "long term care*" OR “residential*”) AND (("Medical Error*" OR "Diagnostic Error*" OR "Medication Error*" OR "drug error*" OR "Observer Variation" OR "Patient safety" OR incident OR incidents OR "adverse event*" OR "near miss" OR "Risk Management" OR "Safety Management" OR Accident OR Accidents OR "Accident Prevention" OR Safety OR complication) AND ("Data Collection" OR "medical record*" OR "Health Record*" OR "report* system*" OR "anonymous report*" OR "non-anonymous report*" OR "voluntary report*" OR reporting)) |
| S6 | (noft("incident report*" OR "error report*" OR "incidents report*" OR "errors report*") AND noft("care home*" OR "nursing home*" OR "long term care*" OR “residential*”) AND (("Medical Error*" OR "Diagnostic Error*" OR "Medication Error*" OR "drug error*" OR "Observer Variation" OR "Patient safety" OR incident OR incidents OR "adverse event*" OR "near miss" OR "Risk Management" OR "Safety Management" OR Accident OR Accidents OR "Accident Prevention" OR Safety OR complication) AND ("Data Collection" OR "medical record*" OR "Health Record*" OR "report* system*" OR "anonymous report*" OR "non-anonymous report*" OR "voluntary report*" OR reporting))) |
| S7 | (noft("incident report*" OR "error report*" OR "incidents report*" OR "errors report*") AND noft("care home*" OR "nursing home*" OR "long term care*" OR “residential*”) AND (("Medical Error*" OR "Diagnostic Error*" OR "Medication Error*" OR "drug error*" OR "Observer Variation" OR "Patient safety" OR incident OR incidents OR "adverse event*" OR "near miss" OR "Risk Management" OR "Safety Management" OR Accident OR Accidents OR "Accident Prevention" OR Safety OR complication) AND ("Data Collection" OR "medical record*" OR "Health Record*" OR "report* system*" OR "anonymous report*" OR "non-anonymous report*" OR "voluntary report*" OR reporting))) – Limits applied from year 2000 |
| S8 | (noft("incident report*" OR "error report*" OR "incidents report*" OR "errors report*") AND noft("care home*" OR "nursing home*" OR "long term care*" OR “residential*”) AND (("Medical Error*" OR "Diagnostic Error*" OR "Medication Error*" OR "drug error*" OR "Observer Variation" OR "Patient safety" OR incident OR incidents OR "adverse event*" OR "near miss" OR "Risk Management" OR "Safety Management" OR Accident OR Accidents OR "Accident Prevention" OR Safety OR complication) AND ("Data Collection" OR "medical record*" OR "Health Record*" OR "report* system*" OR "anonymous report*" OR "non-anonymous report*" OR "voluntary report*" OR reporting))) – Limits applied English Language |
| **SCOPUS** | |
| S1 | TITLE-ABS-KEY(“incident report*" OR "error report*" OR "incidents report*" OR "errors report*") |
| S2 | TITLE-ABS-KEY("care home*" OR "nursing home*" OR "long term care*" OR “residential*”) |
| S3 | (TITLE-ABS-KEY(“incident report*" OR "error report*" OR "incidents report*" OR "errors report*") AND (TITLE-ABS-KEY("care home*" OR "nursing home*" OR "long term care*" OR “residential*”) |
| S4 | TITLE-ABS-KEY("Medical Error*" OR "Diagnostic Error*" OR "Medication Error*" OR "drug error*" OR "Observer Variation" OR "Patient safety" OR incident OR incidents OR "adverse event*" OR "near miss" OR "Risk Management" OR "Safety Management" OR Accident OR Accidents OR "Accident Prevention" OR Safety OR complication) AND TITLE-ABS-KEY ("Data Collection" OR "medical record*" OR "Health Record*" OR "report* system*" OR "anonymous report*" OR "non-anonymous report*" OR "voluntary report*" OR reporting)) |
| S5 | (TITLE-ABS-KEY("care home*" OR "nursing home*" OR "long term care*" OR “residential*”) AND ((TITLE-ABS-KEY("Medical Error*" OR "Diagnostic Error*" OR "Medication Error*" OR "drug error*" OR "Observer Variation" OR "Patient safety" OR incident OR incidents OR "adverse event*" OR "near miss" OR "Risk Management" OR "Safety Management" OR Accident OR Accidents OR "Accident Prevention" OR Safety OR complication) AND TITLE-ABS-KEY ("Data Collection" OR "medical record*" OR "Health Record*" OR "report* system*" OR "anonymous report*" OR "non-anonymous report*" OR "voluntary report*" OR reporting)) |
| S6 | ((TITLE-ABS-KEY(“incident report*" OR "error report*" OR "incidents report*" OR "errors report*") AND (TITLE-ABS-KEY("care home*" OR "nursing home*" OR "long term care*" OR “residential*”)) AND (((TITLE-ABS-KEY("Medical Error*" OR "Diagnostic Error*" OR "Medication Error*" OR "drug error*" OR "Observer Variation" OR "Patient safety" OR incident OR incidents OR "adverse event*" OR "near miss" OR "Risk Management" OR "Safety Management" OR Accident OR Accidents OR "Accident Prevention" OR Safety OR complication) AND TITLE-ABS-KEY ("Data Collection" OR "medical record*" OR "Health Record*" OR "report* system*" OR "anonymous report*" OR "non-anonymous report*" OR "voluntary report*" OR reporting))) |
| S7 | ((TITLE-ABS-KEY(“incident report*" OR "error report*" OR "incidents report*" OR "errors report*") AND (TITLE-ABS-KEY("care home*" OR "nursing home*" OR "long term care*" OR “residential*”)) AND (((TITLE-ABS-KEY("Medical Error*" OR "Diagnostic Error*" OR "Medication Error*" OR "drug error*" OR "Observer Variation" OR "Patient safety" OR incident OR incidents OR "adverse event*" OR "near miss" OR "Risk Management" OR "Safety Management" OR Accident OR Accidents OR "Accident Prevention" OR Safety OR complication) AND TITLE-ABS-KEY ("Data Collection" OR "medical record*" OR "Health Record*" OR "report* system*" OR "anonymous report*" OR "non-anonymous report*" OR "voluntary report*" OR reporting))) AND (LIMIT-TO- (PUBYEAR, 2021) OR (PUBYEAR, 2020) OR (PUBYEAR, 2019) OR (PUBYEAR, 2018) OR (PUBYEAR, 2017) OR (PUBYEAR, 2016) OR (PUBYEAR, 2015) OR (PUBYEAR, 2014) OR (PUBYEAR, 2013) OR (PUBYEAR, 2012) OR (PUBYEAR, 2011) OR (PUBYEAR, 2010) OR (PUBYEAR, 2009) OR (PUBYEAR, 2008) OR (PUBYEAR, 2007) OR (PUBYEAR, 2006) OR (PUBYEAR, 2005) OR (PUBYEAR, 2004) OR (PUBYEAR, 2003) OR (PUBYEAR, 2002) OR (PUBYEAR, 2001) OR (PUBYEAR, 2000) |
| S8 | ((TITLE-ABS-KEY(“incident report*" OR "error report*" OR "incidents report*" OR "errors report*") AND (TITLE-ABS-KEY("care home*" OR "nursing home*" OR "long term care*" OR “residential*”)) AND (((TITLE-ABS-KEY("Medical Error*" OR "Diagnostic Error*" OR "Medication Error*" OR "drug error*" OR "Observer Variation" OR "Patient safety" OR incident OR incidents OR "adverse event*" OR "near miss" OR "Risk Management" OR "Safety Management" OR Accident OR Accidents OR "Accident Prevention" OR Safety OR complication) AND TITLE-ABS-KEY ("Data Collection" OR "medical record*" OR "Health Record*" OR "report* system*" OR "anonymous report*" OR "non-anonymous report*" OR "voluntary report*" OR reporting))) AND (LIMIT-TO (LANGUAGE, “English”)) |
| **Web of Science** | |
| S1 | TS=(“incident* report*" OR "error* report*") |
| S2 | TS=("care home*" OR "nursing home*" OR "long term care*" OR “residential*”) |
| S3 | #2 AND #1 |
| S4 | TS=("Medical Error*" OR "Diagnostic Error*" OR "Medication Error*" OR "drug error*" OR "Observer Variation" OR "Patient safety" OR incident OR incidents OR "adverse event*" OR "near miss" OR "Risk Management" OR "Safety Management" OR Accident OR Accidents OR "Accident Prevention" OR Safety OR complication) AND TS=("Data Collection" OR "medical record*" OR "Health Record*" OR "report* system*" OR "anonymous report*" OR "non-anonymous report*" OR "voluntary report*" OR reporting) |
| S5 | #4 AND #2 |
| S6 | #5 OR #3 |
| S7 | #5 OR #3  Refined by: [excluding] PUBLICATION YEARS: (1997 OR 1996 OR 1995 OR 1994 OR 1993 OR 1992 OR 1999 OR 1991 OR 1998) |
| S8 | #5 OR #3  Refined by: [excluding] PUBLICATION YEARS: (1997 OR 1996 OR 1995 OR 1994 OR 1993 OR 1992 OR 1999 OR 1991 OR 1998) AND LANGUAGES: (ENGLISH) |
| **Grey Literature – MEDNAR and OpenGrey** | |
| S1 | (("care home*" OR "nursing home*" OR "long term care*" OR “residential*”) AND (("Medical Error*" OR "Diagnostic Error*" OR "Medication Error*" OR "drug error*" OR "Ob-server Variation" OR "Patient safety" OR incident OR incidents OR "adverse event*" OR "near miss" OR "Risk Management" OR "Safety Management" OR Accident OR Accidents OR "Accident Prevention" OR Safety OR complication ) AND ("Data Collection" OR "medical record*" OR "Health Record*" OR "report* system*" OR "anonymous report*" OR "non-anonymous report*" OR "voluntary report*" OR reporting)) ) OR ( ("care home*" OR "nursing home*" OR "long term care*" OR “residential*”) AND (("Medical Error*" OR "Diagnostic Error*" OR "Medication Error*" OR "drug error*" OR "Ob-server Variation" OR "Patient safety" OR incident OR incidents OR "adverse event*" OR "near miss" OR "Risk Management" OR "Safety Management" OR Accident OR Accidents OR "Accident Prevention" OR Safety OR complication) AND ("Data Collection" OR "medical record*" OR "Health Record*" OR "report* system*" OR "anonymous report*" OR "non-anonymous report*" OR "voluntary report*" OR reporting))) |
